# Conceptualizing the commercial determinants of dietary behaviors associated with obesity: A systematic review using principles from critical interpretative synthesis

**DOI:** 10.1002/osp4.507

**Published:** 2021-04-05

**Authors:** Yanaina Chavez‐Ugalde, Russell Jago, Zoi Toumpakari, Matt Egan, Steven Cummins, Martin White, Paige Hulls, Frank De Vocht

**Affiliations:** ^1^ National Institute for Health Research School for Public Health Research Newcastle upon Tyne UK; ^2^ Population Health Sciences, Bristol Medical School University of Bristol Bristol UK; ^3^ Centre for Exercise, Nutrition & Health Sciences, School for Policy Studies University of Bristol Bristol UK; ^4^ National Institute for Health Research Collaboration for Leadership, Applied Health Research and Care West (NIHR CLAHRC West) Bristol UK; ^5^ Department of Public Health, Environments and Society London School of Hygiene and Tropical Medicine London UK; ^6^ Centre for Diet and Activity Research (CEDAR) MRC Epidemiology Unit University of Cambridge Cambridge UK

**Keywords:** commercial determinants of obesity, dietary behavior, food industry, public health

## Abstract

**Introduction:**

Unhealthy diet is an important preventable risk factor for overweight and obesity. Identifying the key drivers of an unhealthy diet is an important public health aim. “Big Food” has been identified as an influential factor shaping dietary behavior and obesity, and their practices have broadly been labeled as the “commercial determinants of obesity,” but there is a lack of definitions and conceptualizations for these terms. This review aimed to synthesize literature on the commercial determinants of dietary behavior associated with obesity. It presents the development of an integrative definition and a conceptual framework involving potential influences on dietary behavior, and it examines the prevalence of certain narratives within papers that focus on children and adolescents.

**Methods:**

Four electronic databases (Ovid MEDLINE, PubMed, Web of Science, and Scopus) were searched up to December 2020. Eighty‐one articles met the inclusion criteria: they were published in a peer‐reviewed academic journal, described a practice from the food/beverage industry in relation to dietary behavior or obesity. Data were integrated using critical interpretative synthesis.

**Results:**

The commercial determinants of dietary behavior are conceptualized in terms of three corporate spheres of action—*political and legal; production, processing and design; and marketing and preference shaping*—which enable powerful food industry to successfully pursue their business, market, and political objectives. The most frequently reported sphere of action targeting children and adolescents was *marketing and preference shaping*.

**Conclusions:**

In the included literature, the commercial determinants of dietary behavior associated with obesity have been conceptualized as being part of a complex system where corporate practices are enabled by power structures. The proposed framework can facilitate a structured identification and systematic study of the impact of specific aspects of food industry's strategies and increase opportunities for primary prevention by anticipating industry responses and by discouraging corporate practices that harm health.

## 1 INTRODUCTION

The global increase in obesity is associated with the increased availability and consumption of energy‐dense, nutrient‐poor foods and beverages, many of which are “ultra‐processed.”^1^, ^2^, ^3^ A key contributing factor is the continuing expansion and concentration of power of transnational food and beverage corporations (“Big Food”/food industry).^4^, ^5^ About 75% of the global food sales include processed foods, for which the largest producers hold over a third of the global market.^6^, ^7^, ^8^ This has led to an accelerated “nutrition transition” from more traditional diets to highly processed foods.^9^, ^10^

Many authors have suggested that food industry practices have shaped the eating environment and determined food choices^11^ through food availability, pricing, social, and cultural desirability.^12^, ^13^, ^14^ Factors related to the food system that promote obesogenic dietary behaviors have broadly been labeled as the “commercial determinants of obesity.”^15^, ^16^

The term “commercial determinants of health” (CDoH) was first used by West and Marteau,^17^ who defined it as: “Factors that infiuence health which stem from the profit motive.” Millar proposed the term “corporate determinants of health”^18^ to describe how companies can act in ways that benefit society, but also how they can have negative influences on population health. In 2016, Kisckbusch et al.^19^ further defined the term as “strategies and approaches used by the private sector to promote products and choices that are detrimental to health” and conceptualized health outcomes as being determined by the influence of corporate activities on the cultural and social environments. In 2018, Madureira‐Lima and Galea presented^20^ and applied^21^ a framework to map corporate practices and its impact on health, and conceptualized power as the vehicle through which corporations exert their influence on preference shaping and on the political, knowledge, legal, and extra‐legal environments.

Although there is a developing discussion on the commercial determinants as drivers of ill‐health, there has not been a comprehensive review that conceptualizes and defines these factors and the ways in which they can directly and indirectly influence dietary behavior and obesity.

Two systematic reviews^22^, ^23^ and an overview^24^ of the CDoH were recently published, showing that corporations use market (i.e., commodities themselves and production practices) and nonmarket practices (e.g., extensive supply chains, corporate political activities) to sell their products and secure a favorable regulatory environment,^22^, ^24^ as well as that the role of commercial actors as drivers of ill‐health are frequently obscured, understated or simply absent in the existing frameworks of the determinants of health.^23^

The current systematic review extends the work in previous reviews by addressing four specific, focused aims: (1) synthesize the literature on the commercial determinants of dietary behavior associated with obesity, (2) develop an integrative definition of this concept, specific to the food and beverage industry, (3) develop a conceptual framework of food industry activities that unintendedly undermine nutrition globally, and (4) examine the prevalence of certain narratives in the selected academic literature and within papers that focus on children and adolescents.

## 2 METHODS

The Cochrane Handbook for Systematic Reviews of Interventions^25^ guided the methodology for this review. Risk of bias was assessed using Cullerton et al.'s^26^ key guiding principles for population health researchers working with food industry. Principles from critical interpretative synthesis (CIS) were used to guide the data synthesis^27^ and iteratively refine the research questions while searching and selecting from the literature. CIS allows integrating and interpreting a substantial body of data from different types of research evidence and across multi‐disciplinary fields into a coherent conceptual framework (“synthesising argument”), grounded in the concepts identified in the included articles. The review protocol was registered with PROSPERO, registration number CRD42019137363.

### 2.1 Search strategy

Searches were developed between April and June 2019, and updated in December 2020, to identify relevant literature published in peer‐reviewed journals on the commercial determinants of dietary behaviors associated with obesity. Systematic searches were done in the following databases: MEDLINE (Ovid), PubMed, Web of Science, and Scopus from inception and with no restriction on date or country of publication. Only documents written in English or Spanish were included. Keyword searches included: [(commercial OR corporate).mp AND (determinant*).mp)] AND [(food OR drink).mp AND (industry*.mp)] AND [(diet* behav* OR food choice* OR dietary intake OR nutrition* OR eating behav*).mp] OR (obes* OR overweight OR health).mp]. Database and reference search with results can be found in Section 1 of the Supporting Information Data S1. In addition to database searches, experts were asked for any other relevant documents for inclusion. Reference lists of included documents were hand searched to find any additional eligible articles.

#### 2.1.1 Inclusion criteria

Articles had to fulfill the following criteria: published in a peer‐reviewed journal (including commentary pieces), books or book sections; written in English or Spanish; refer to humans; propose a definition, or describe a mechanism (e.g., influencing policymakers to maintain a business‐friendly regulatory environment), framework, or practices (e.g., lobbying) from the food and/or beverage industry that relates to their commercial or corporate activities in relation to dietary behavior and/or its link with health or obesity; refer to food and beverage industry exclusively.

### 2.2 Article screening

Database searches were managed using EndNote X9 and screened using Rayyan QCRI. One author (Y.C.U.) retrieved and screened titles for eligibility. Y.C.U. and a second reviewer (P.H.) screened titles and abstracts selected after the first title screening. Full text screening was done independently by the two reviewers. Reasons for exclusion were documented and discussed until agreement was reached.

### 2.3 Data extraction

Data extraction was done by Y.C.U. and reviewed by Y.C.U., Z.T., R.J., and F.D.V. Data were extracted for author and year, article name, publication type, country/region, income level, field of study, population age group, mention of adolescents (10–19 years), health outcome, definition or mechanism, conflict of interest reported, and details for potential conflict of interest. To capture the whole range of definitions, key terms, and mechanisms that have been used in the academic literature, there was no restriction on age groups. Once the final list of included articles was selected, a subgroup of articles was created that focused on adolescence (10–19 years). For the purposes of this review, if the article referred to “youth” or “young people” without specifying age, they were considered to be ≤19 years old and were included as part of the “adolescent” group to identify the dominant narratives and most frequently mentioned practices targeting this age group.

### 2.4 Quality assessment and risk of bias

Risk of bias and other aspects of study quality were not assessed since CIS recommends prioritizing relevant articles rather than selecting study types.^28^ Conflicts of interest (CoI) reported by the authors were documented, discussed, and explored using Cullerton et al.'s guiding principles to help identify, prevent, and manage actual or perceived CoI. Even if authors reported not to have any CoIs or competing interests, these guiding principles still enabled critical appraisal of potential CoIs. For example, reporting not having competing interests but being employed by the food industry or by an industry funded organization could influence study findings.^29^, ^30^, ^31^, ^32^ If any potential CoIs were identified, findings from the study were critically appraised to identify if, for example, the study funding source or collaboration with food industry could have influenced the study results.

### 2.5 Data synthesis

Guided by CIS^27^ principles, data synthesis covered the following steps. Fragments of text that made reference to corporate activities in relation to dietary behavior and obesity were extracted and coded by the first author. A second researcher (Z.T.) independently double‐coded thirty percent of the included articles, and any discrepancies were discussed until agreement was reached. Codes that explained similar ideas were iteratively grouped into themes and subthemes. Based on the themes and subthemes, authors developed higher‐level conceptual themes (data‐driven themes). The relationship between the data‐driven themes, themes, and subthemes enabled the creation of the conceptual framework showing how the commercial determinants of diet and obesity operate.

## 3 RESULTS

### 3.1 Search results

**FIGURE 1:**
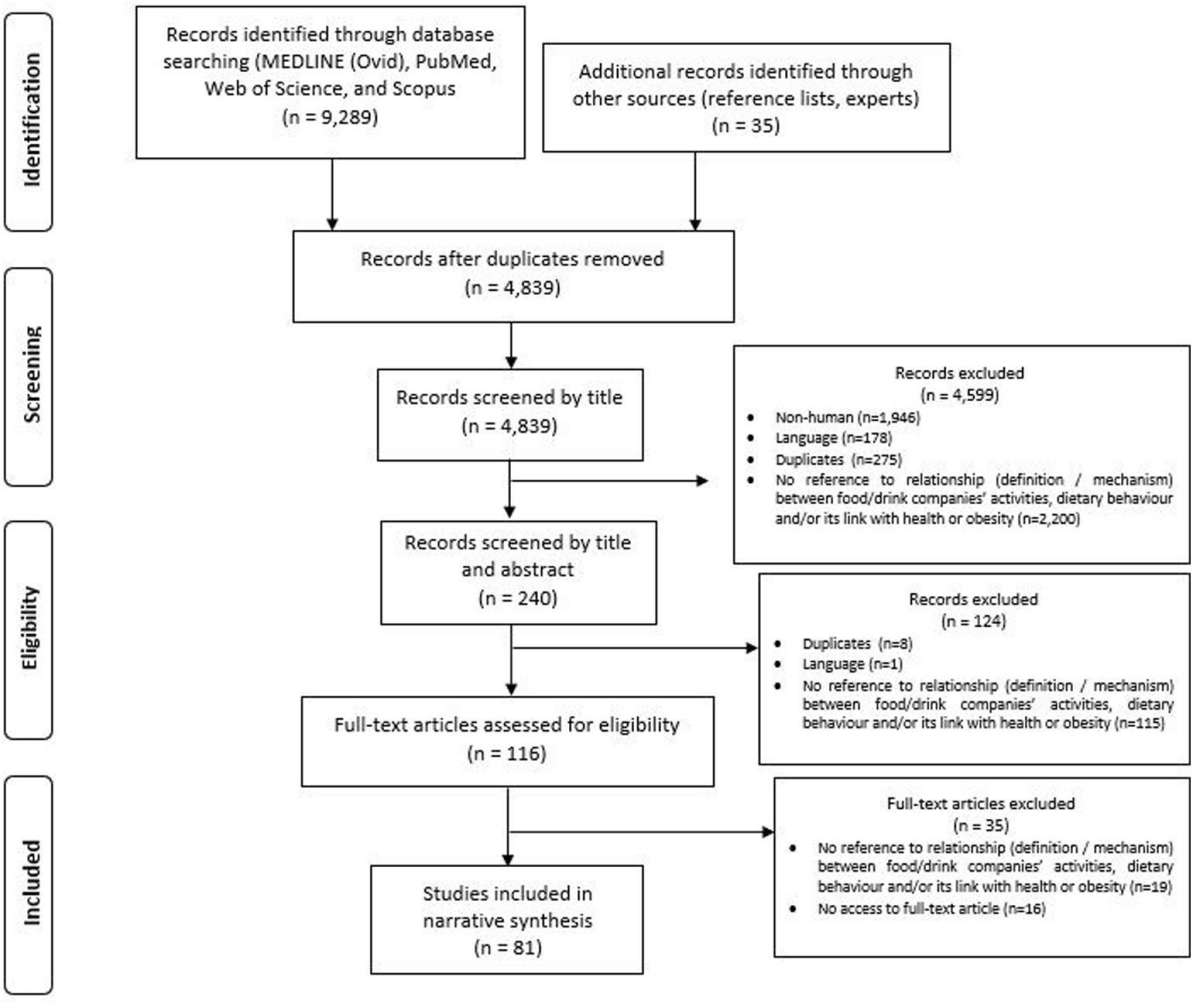
PRISMA flow diagram

A PRISMA flow diagram (Figure 1) documents the search, screening, and selection process of the 81 included articles.

### 3.2 Descriptive information

The largest number of identified studies focused on high income countries (*n* = 37; 46%). The field of study was mainly focused on public health (*n* = 51; 63%) and health policy (*n* = 17; 21%), followed by nutrition (*n* = 3; 4%), law (*n* = 2; 2%), business (*n* = 2; 2%), anthropology (*n* = 1; 1%), and sociology (*n* = 1; 1%). Only 23 articles (28%) referred to adolescents (10–19 years) with the majority (*n* = 58; 72%) not specifying an age group. About half of the studies (*n* = 38; 47%) focused on obesity, while 32 articles (40%) focused on diet related noncommunicable diseases. Potential CoIs were found in four articles (5%),^33^, ^34^, ^35^, ^36^ but three of those took measures to explicitly manage these,^33^, ^35^, ^36^ for example, limiting the involvement of the funder in any aspects of the project^33^, ^36^; explicitly reporting the nature of funding received from the food industry^33^, ^35^, ^36^; and including findings that were unfavorable to the funder.^33^, ^35^, ^36^ The conclusion drawn from these studies was that there were no CoIs since, even when being employed by the food industry or by an industry funded organization, the funding source or collaboration with the food industry should not have had an influence on study findings and results did not seem to enhance industry's reputation or influence over the evidence base of diet and obesity. In contrast, in one study^34^ the author was employed, and the study was funded, by the food industry, but these were not reported as this being a potential CoIs, and study findings and results seemed to only enhance industry's reputation. A detailed table with descriptive information on the 81 included articles and details on CoI can be found in Tables S2 and S3. Extracted quotes of definitions and mechanisms found in the 81 articles included can be found in Table S4.

### 3.3 A conceptual framework for the commercial determinants of dietary behavior and obesity

**FIGURE 2:**
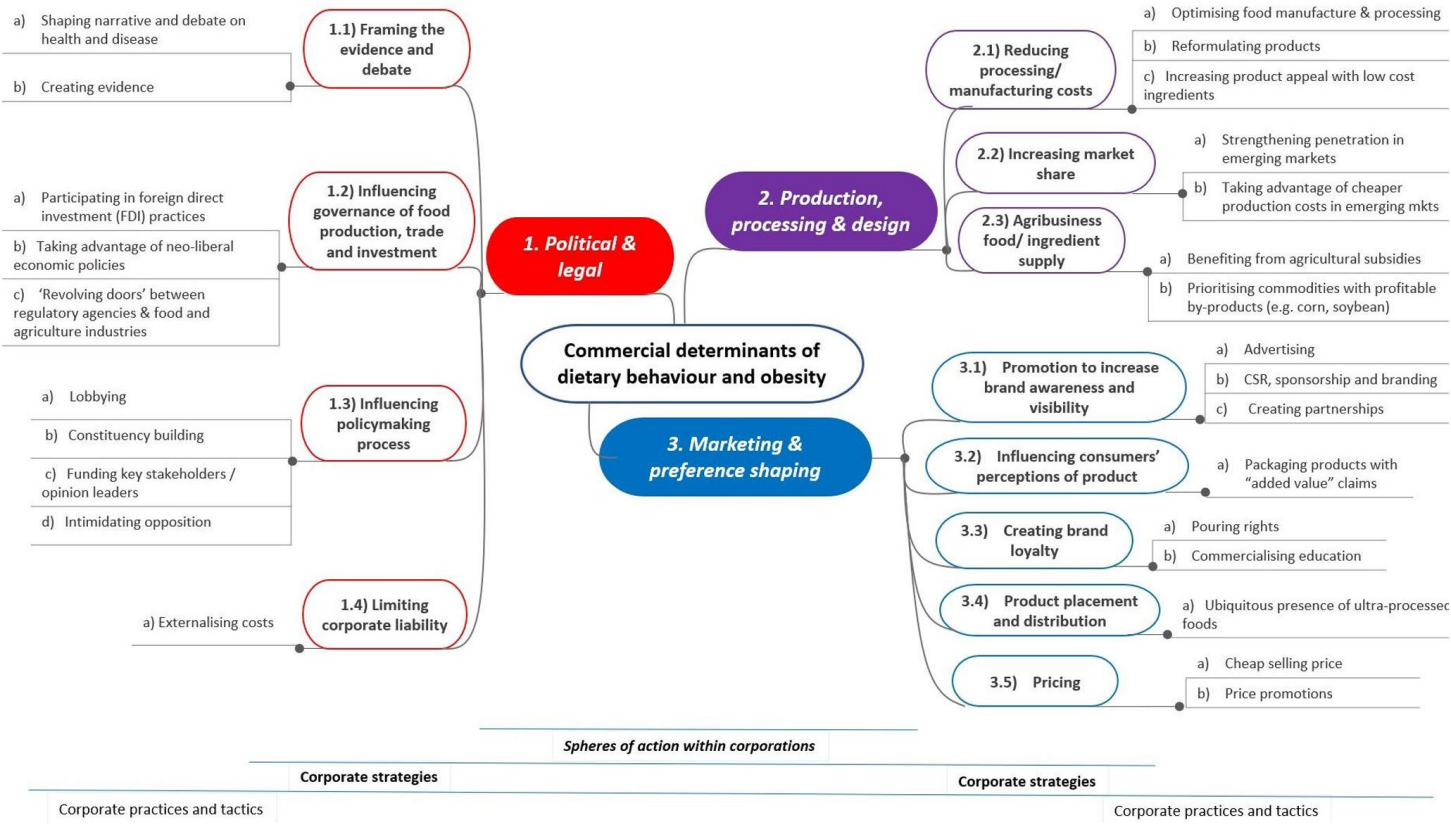
Visual representation of the framework for the commercial determinants of dietary behaviors and obesity

Three data‐driven themes were developed and fell under the concept of “food industry's spheres of action”: *1*. *political and legal; 2. production, processing and design; and 3. marketing and preference shaping*. The framework was developed by expanding on these three data‐driven themes and resulted in 12 themes (“corporate strategies”), 26 subthemes (“corporate practices”), and 85 mechanisms. A table with details on the themes, subthemes, and mechanisms can be found in Table S5. A visual representation of the framework (i.e., the relationship between themes and subthemes) is presented in Figure 2.

#### 3.3.1 Sphere of action 1: Political and legal

The political and legal sphere of action aims to generate a business‐friendly regulatory and discursive environment. To achieve this, corporations employ a range of strategies and practices. Four corporate strategies (themes) and 10 practices (subthemes) were identified.

##### Strategy 1.1: Framing evidence and debate

Through this strategy, the food industry aims to frame the evidence and debate of diet and obesity as an issue of individual and societal choices and responsibilities.^37^, ^38^, ^39^, ^40^ This framing has the potential to shift the focus away from dietary behavior (e.g., emphasizing physical activity over diet and calorie intake on obesity) and limit the perception that policymakers have on the food industry's responsibility for the products they produce, promote, and sale, particularly ultra‐processed foods which have been linked with excess calorie intake and weight gain^41^. This strategy is enacted by shaping narrative and debate of health and disease and through the creation of evidence.

*Practice 1.1 (a): Shaping narrative and debate of health and disease*. This was a commonly reported practice which allowed food‐related corporate interests to shift focus away from health and reframe regulatory efforts (e.g., soft drink taxes) as an issue of consumer rights and to highlight these efforts as a restriction to people's freedom of choice.^15^, ^24^, ^42^, ^43^, ^44^, ^45^, ^46^, ^47^, ^48^, ^49^, ^50^

(…) food industry selectively produces and disseminates information that would be beneficial to its activities, to infiuence public policies and public opinion in ways favourable to its companies ^48^

*Practice 1.1 (b): Creating evidence*. By funding research, conferences, creating evidence for obesity causes and solutions, industry can create a body of literature and supportive narratives that maintain doubt and detract attention from the health implications of consumption of their products.^40^, ^45^, ^51^, ^52^, ^53^

The strategies (…) that unhealthy commodity industries use to promote their products and choices that are detrimental to health (…) include influencing the creation of evidence.^54^

Food industry tactics to influence policy: (…) creation or funding of alliances or front groups; funding research to create or maintain doubt about health implications.^55^

##### Strategy 1.2: Influencing governance of food production, trade, and investment

Global food industry firms benefit from, and aim to maintain, a global liberalized trading environment where their corporate and economic power can shape the landscape of the food environment and nutrition worldwide and limit the effectiveness of traditional governance mechanisms.^56^ This is made possible by participating in foreign direct investment (FDI) practices, by taking advantage of neoliberal economic policies that favor trade liberalization and globalization, and through benefiting from agricultural export subsidies.

*Practice 1.2 (a): Participating in foreign direct investments (FDIs)*. Corporate economic and political power allows the global food industry to have unrestricted capital flows in emerging markets, grow through mergers, and joint ventures. This allows them to have an increased control over different levels of the food system (production, processing, distribution, retail).^20^, ^55^, ^57^, ^58^, ^59^, ^60^

Transnational food companies powerfully shape the supply, demand, and consumption of food and beverage products. (...) Transnational food companies are moving quickly into markets in developing countries, using strategies such as foreign direct investment to increase production and sales.^58^

*Practice 1.2 (b): Taking advantage of neoliberal economic policies*. Neoliberal policies that enable the opening of trading markets have allowed a widespread distribution of commodities that are conducive to the production and distribution of ultra‐processed foods and sugary beverages.^22^, ^59^, ^60^, ^61^

(…) neoliberalism, an ideology that favors deregulation, privatization, and the supremacy of markets, has strengthened the power of corporations and weakened the role of government in public health regulation.^62^

*Practice 1.2 (c): “Revolving doors” between regulatory agencies & food and agriculture industries*. There are national agencies whose remit includes setting the governance rules of food production, trade, and investment. Officials from these regulatory agencies are sometimes recruited from food industry and agribusinesses (or vice versa), and in some cases then move on to become lobbyists in favor of the food industry interests.^5^ This becomes a “revolving door” between public and private sectors that gives key access to decision makers and valuable knowledge and relationships that allows them to shape governance systems.^5^, ^21^, ^39^, ^63^, ^64^, ^65^

There is a long history of USDA leaders and leaders of other agencies being recruited from food and agriculture industries and then returning to businesses like lobbying firms when their government service ends.^5^

##### Strategy 1.3: Influencing policymaking process

Influencing the policymaking process was the most frequently mentioned strategy.^22^, ^35^, ^38^, ^39^, ^42^, ^43^, ^45^, ^46^, ^50^, ^51^, ^56^, ^63^, ^65^, ^66^, ^67^, ^68^, ^69^, ^70^, ^71^, ^72^ Food industry have been able to influence policy and governance through market dominance, which has given them power to influence policy agendas.

Within this strategy, four corporate practices were identified.

*Practice 1.3 (a): Lobbying*. Lobbying was the most reported practice within influencing the policymaking process strategy.^18^, ^35^, ^42^, ^48^, ^51^, ^52^, ^62^, ^63^, ^71^, ^73^, ^74^, ^75^ Shaping the regulatory environment is critical to corporate profits^52^ and lobbying was identified as a practice through which corporations exert their power to maintain a business‐friendly regulatory environment.^19^, ^20^, ^24^, ^62^, ^65^

Through lobbying (…) they [“Big Food”] have directly sought to infiuence policy and governance.^63^

*Practice 1.3 (b) Constituency building*. Through constituency building, food industry seeks to get involved in the community, establish relationships with key stakeholders and highlight media and public opinion that support industry's position.^45^, ^65^, ^68^ This practice includes philanthropic activities, promoting public–private partnerships and public relationships to recruit supporters and detract opposition.^50^, ^65^, ^68^

Constituency building… attempts to infiuence public opinion and public policies and programmes.^48^

*Practice 1.3 (c): Funding key stakeholders/opinion leaders*. Giving financial incentives to key stakeholders and opinion leaders (e.g., election campaigns, health and nutrition organizations, opposition groups) creates a supportive environment for food industry activities and helps to maintain a business‐friendly regulatory environment.^36^, ^39^, ^51^, ^76^

“(…) corporations penetrate all aspects of society, from macrosocial and political aspects, such as corporate donations to election campaigns.”^21^

*Practice 1.3 (d): Intimidating opposition*. By intimidating opponents, the food industry aims to disrupt activities that have the potential to negatively impact on their business. These activities include posing legal threats against public policies and industry opponents,^45^ intimidating scientists by creating doubt about their integrity and their work^35^ and by using media leverage to criticize public health advocates.^31^

Food industry threatened to litigate against potential government policy through legal channels (…) industry aimed to intimidate policy makers by citing potential barriers to free trade if such policy was introduced.^45^

##### Strategy 1.4: Limiting corporate liability

By limiting their corporate liability, the food industry can limit the extent to which they can be held accountable for their activities which are harmful to health.^56^, ^67^, ^77^, ^78^

*Practice 1.4 (a): Externalizing costs*. The food industry has implemented practices to externalize costs using unregulated areas of activity, such as keeping prices artificially low by outsourcing sectors of their business^79^ and shifting profits to tax havens.^24^, ^73^ These corporations have the power to keep the price of harmful products artificially low with the final price not reflecting the full true cost of production and the costs of the damage caused by the consumption of their products.^80^

(…) there is externalization of costs to the public from profit‐shifting, tax‐havens, and service fees paid back to USA headquarters. (…) The health costs of non‐communicable diseases and environmental impacts from McDonald's operations are externalized to the community.^73^

#### 3.3.2 Sphere of action 2: Production, processing, and design

The production, processing, and design corporate sphere of action aims to optimize cost viability. To achieve this, corporations employ two corporate strategies (themes) and five practices (subthemes).

##### Strategy 2.1: Reducing processing/manufacturing costs

The food industry can reduce production costs using optimization practices (e.g., mass production and economies of scale),^64^, ^73^, ^79^, ^81^, ^82^, ^83^, ^84^ reformulating and manufacturing products with low‐cost ingredients that enhance palatability (e.g., fat, sugar, salt, caffeine).^57^, ^59^, ^67^, ^85^, ^86^

*Practice 2.1 (a): Optimizing food manufacture and processing*. Technological advancement and the usage of economies of scale has enabled massive manufacturing and processing of energy‐dense/low nutritional value foods.^80^, ^87^, ^88^ These foods are highly palatable, attractive to the consumer due to their convenience for purchase, and consumption and cheaper to produce.^81^, ^84^

Large food, beverage (…) firms are among the most internationalised businesses in the entire economy. (…) Economies of scale are an important factor in the profitability of food, wholesale, retail, and beverage firms (…).^87^

*Practice 2.1 (b): Reformulating*. Reformulating products can serve two purposes; (1) increase the ratio of cheap ingredients to reduce production and processing costs and (2) serve the discursive purpose that industry, is part of the solution.^57^

Changing product recipes may be good brand protection but has little population dietary impact (…). They favor a technical approach to nutrition to justify the products they produce and sell.^57^

*Practice 2.1 (c): Increasing product appeal with low‐cost ingredients*. Products high in fat, sugar and/or salt have a high sensory appeal, increase shelf‐life, and by being cheap, generate large profit margins, especially with high‐volume sales. These ingredients are commonly used in high proportions to manufacture energy‐dense and ultra‐processed foods.^35^, ^89^

“Obesogenic” food companies maximize their profits by maintaining or increasing sales and prioritizing both types of addictive mechanism (e.g. “value deals” and addictive properties of sugar, salt, fat and caffeine on foods).^86^

##### Strategy 2.2: Increasing market share

Increasing food industry's market share is both an outcome and a driver for commercial profit‐making strategies and practices. Increased corporate growth due to sales and profit‐margins increases corporate power which allows continued market penetration in emerging markets and enables them to take advantage of cheaper production costs while continuously optimizing their production and processing costs.^24^, ^62^, ^65^, ^74^, ^90^, ^91^, ^92^

*Practice 2.2 (a): Strengthening penetration in emerging markets*. By extending their corporate dominion, food industry has become richer and more powerful.^90^ Penetrating emerging markets has been a key practice used to continue growing and expanding their business strategies (e.g., diversifying their product portfolio) and design products according to local offering of ingredients and demand (e.g., glocalisation).^54^, ^58^, ^84^

(…) low‐ and middle‐income countries, have been identified as emerging markets for Big Food.^37^

Breadth and depth of corporate influence is expanded as more people are reached with ever more consumption choices.^19^

*Practice 2.2 (b): Taking advantage of cheaper production costs in emerging markets*. Having access to a variety of markets allows corporations to decide where to establish their manufacturing plants and where to get their supply of ingredients from (e.g., bulk buying of local commodities at lower prices and settling production and processing plants in places where labor costs are cheaper).^59^, ^60^, ^86^

(…) the economic causes of under‐nutrition and over‐weight have a common structural basis, driven by multinational corporations' demand for cheap labour and new consumers.^86^

##### Strategy 2.3: Agribusiness food/ingredient supply

The food value chain begins with the production input (i.e., materials for crop production and seeds), followed by farmers, growers, and agribusinesses that provide raw agricultural commodities. Therefore, the nutritional quality of the food environment is strongly influenced by the ingredients that the food and beverage industry use to manufacture their products.^69^ Additionally, which and how much of these ingredients are produced is determined by regulations and targets set for agricultural production, economic performance, and competitiveness for agribusinesses.^5^, ^21^, ^46^, ^63^, ^64^, ^69^, ^80^, ^81^, ^93^

*Practice 2.3 (a): Benefiting from agricultural subsidies*. Agricultural export subsidies have encouraged conversion of traditional domestic production to export‐oriented production^60^ or cash‐crops and have prioritized commodities with highly profitable by‐products (e.g., corn, soybean).^55^, ^71^, ^79^

“...dietary shift is also attributed to the continued agricultural export subsidies that allow developed countries to artificially suppress food prices making it difficult for domestic markets in developing countries to complete.”^55^

*Practice 2.3 (b): Prioritizing commodities with profitable by‐products (e.g., corn, soybean)* 1. Food and beverage industry use ingredients that will maintain the essential composition of their products, but equally, keep their processing costs within their budget and keep prices stable at retail point. Simultaneously, governments set regulations for commodity production and economic performance to maintain production at competitive market level,^69^ which becomes an incentive to prioritize the production of commodities with higher productivity and that will generate higher profits.^79^ Such is the case of soybeans and corn.^94^ With increased productivity, the price of these commodities reduces.^79^ Although this can increase farmers' profits momentarily, eventually, this surplus will result in a reduction in prices. This has two effects: first, food industry can have continuous access to cheap ingredients, making food manufacturers prioritize these ingredients over others; and second, the need to find new applications for corn, soy, and their by‐products. Today, most ultra‐processed food contains some form of corn or soy.^94^

…the low cost of high calorie foods with little nutritional value is due, in part, to federal subsidies for production of corn and soybeans.^79^

#### 3.3.3 Sphere of action 3: Marketing and preference shaping

The marketing and preference shaping corporate sphere of action aims to increase brand loyalty and enhance consumers' desire for their product. To achieve this, five corporate strategies (themes) and nine practices (subthemes) were identified.

##### Strategy 3.1: Promotion to increase brand awareness and visibility

To increase sales of their products, food industry needs to increase brand awareness and visibility in targeted populations.^68^, ^81^ This can be achieved through various channels and strategies including integrated marketing and advertising^73^, ^88^; corporate social responsibility (CSR),^68^ sponsorship, and branding^18^, ^73^, ^95^; and by creating public‐partnerships with key stakeholders, opinion leaders, and influential people that will promote their brand enabling wide visibility and reach.^15^, ^36^, ^54^, ^63^

*Practice 3.1 (a): Advertising*. With technology improvement advertising has become more specialized and sophisticated, and is one of the main and most frequently reported practice food industry has used to attract new consumers, particularly young people, to encourage consumption of their products.^18^, ^21^, ^22^, ^24^, ^33^, ^36^, ^46^, ^58^, ^67^, ^73^, ^74^, ^84^, ^85^, ^86^, ^88^, ^96^, ^97^

[The] food environment and exposure to childhood advertising are important causes of childhood obesity (…).^67^

*Practice 3.1 (b): CSR, sponsorship, and branding*. This was the most frequently mentioned practice to increase brand awareness and visibility.^16^, ^18^, ^36^, ^52^, ^68^, ^73^, ^78^, ^95^, ^96^, ^98^ Through this set of strategies, corporations attempt to obscure the boundary between profit‐making and philanthropic activities.^39^

CSR as primarily a public relations strategy designed to achieve ‘‘innocence by association’’ (…) soda industry CSR aims to position the companies, and their products, as socially acceptable rather than contributing to a social ill.^95^

*Practice 3.1 (c): Creating partnerships*. The food industry advocates to create partnerships with government (e.g., public–private partnerships) highlighting that these can create unique opportunities to leverage effective and more wide‐reaching interventions.^24^, ^69^ However, it is unclear how public interests can be protected and prioritized over commercial interests.

Some critics warn that any partnership creates benefit for industry but see no clear, established or legitimate mechanism through which public health would be protected.^15^

##### Strategy 3.2: Influencing consumers' perceptions of products

Food industry aims to influence consumers behavioral motivations.^86^ Resulting patterns of consumption are influenced by consumers' perception of products, beyond the product itself, and can be heavily influenced by commercial companies, involving developing a brand image that is linked to emotional triggers and convenience.^68^, ^85^, ^86^

*Practice 3.2 (a): Packaging products with “added value” claims*. Focusing on a particular nutrient and labeling it as an “added value” health claim, generating brand differentiation to distinguish one line of products from another, and adding toys and appealing characters can elicit consumers desire for these products.

Health claims allow for a description of the relationship between a food product and its role in disease prevention. Food labelling is a significant marketing tool because of its impact on consumer confidence in food quality and the role it plays in the general discourse of diet and health ^52^

##### Strategy 3.3: Creating brand loyalty

Creating brand loyalty is key for the food industry to ensure consumers consistently purchase their products,^46^, ^85^, ^93^ and they are particularly interested in forging long‐lasting relations with children and adolescents to ensure brand loyalty.^85^

*Practice 3.3 (a): Pouring rights*. A reported practice identified was pouring rights contracts in schools and sports stadiums.^93^, ^99^, ^100^ This practice involves acquiring exclusive permission for a beverage manufacturer or bottler to control distribution and sales in a venue.

(…) exclusive rights to sell one brand are the latest development in the increasing commercialization of school food. These contracts, intended to elicit brand loyalty among young children who have a lifetime of purchases ahead of them.^93^

*Practice 3.3 (b) Commercializing education*. Schools have been used as a marketing venue for the food industry since they can acquire access to a captive audience of young consumers.^18^, ^85^, ^93^, ^97^, ^99^ This includes, for example, corporate creation/sponsorship of curriculum and educational materials,^99^ appropriation of space by sponsoring infrastructure in exchange for branding sports facilities, lunchrooms, or scoreboards.^97^

[A] trend is the intensified focus on schools as marketing venues for corporations (…), including salty snacks, fat‐laden foods, and sugary soft drinks.^99^

##### Strategy 3.4: Product placement and distribution

Food industry has benefited from neoliberal policies and have managed to attain global presence by making use of extended supply chains and distribution channels, and by making ultra‐processed foods increasingly available, accessible, and convenient for consumption worldwide. This was a commonly reported strategy.^16^, ^24^, ^54^, ^59^, ^74^, ^82^, ^83^, ^84^, ^91^, ^101^

*Practice 3.4 (a): Ubiquitous presence of ultra‐processed foods*. Ultra‐processed foods can be found almost everywhere at any time in urban and in high‐ and middle‐income countries.^74^ This combined with industry's marketing practices contributes to excessive consumption of ultra‐processed foods.^33^, ^62^, ^74^, ^80^, ^89^

The combination of high levels of promotion, widespread availability and low prices of these products (...) overwhelmingly drive the behaviours in the direction of positive energy balance.^80^

##### Strategy 3.5: Pricing

Corporations also shape our environments by establishing the pricing strategies for their products, for example, cheap selling price at point of purchase,^24^, ^84^ discounts, bundle deals, price promotions, and coupons and reward programs.^16^, ^62^, ^73^, ^74^, ^75^, ^81^ Consumption patterns are strongly dependent on price.^102^

*Practice 3.5 (a): Cheap selling price*. One factor that influences the consumption of ultra‐processed foods is their cheap selling price.^58^, ^80^, ^86^, ^103^

Transnational food companies (…) are one of the main drivers of the increasing consumption of ultra‐processed foods and sugary beverages, which are cheap, highly palatable, and sold in large portion sizes.^55^

*Practice 3.5 (b): Price promotions*. Price promotions influence consumer's purchasing behavior by delivering more product for the same amount of money, in turn influencing consumer's purchasing preference.^54^, ^79^, ^86^, ^93^, ^97^, ^103^

Food and beverage marketers' and fast‐food restaurants' ongoing marketing and sales promotion efforts, such as value pricing, psychological pricing, quantity discounts, and combo deals, which undermine portion control and healthy food choices.^79^

### 3.4 Commercial determinants of dietary behavior and obesity in children/adolescents

Only 23 of the 81 (28%) articles mentioned anything specific for children/adolescents (≤19 years). There was not a single study focusing on adolescents^10^, ^11^, ^12^, ^13^, ^14^, ^15^, ^16^, ^17^, ^18^, ^19^ exclusively. Most of the commercial determinants of dietary behavior and obesity in children/adolescents are similar compared to the ones for all age groups. However, some aspects are mentioned more frequently, while some others are not mentioned at all, when referring to this age group. The most frequently mentioned corporate strategies for children/adolescents were within the *marketing and preference shaping* sphere of action, namely, *advertising* through targeted and integrated marketing^22^, ^24^, ^33^, ^36^, ^46^, ^58^, ^67^, ^73^, ^84^, ^85^, ^86^, ^88^, ^97^ and *CSR, sponsorship and branding* of sports, cultural events, and educational materials.^22^, ^24^, ^36^, ^68^, ^73^, ^91^, ^93^, ^95^, ^100^, ^101^ The second most reported sphere of action was *production, processing, and design*, in particular, increasing product appeal through low‐cost/addictive ingredients.

### 3.5 Development of an integrative definition for the commercial determinants of dietary behavior associated with obesity

The inductive process of merging overlapping concepts across the included articles allowed to make broader analytic statements about how the commercial determinants of dietary behavior and obesity operate. The patters of meaning and the relationships found between the themes and subthemes allowed for the creation of an integrative definition for the commercial determinants of dietary behavior associated with obesity. The following two‐part definition is therefore proposed:

The commercial determinants of dietary behaviour and obesity are strategies used by the food industry to create a favourable regulatory and discursive environment in which they can produce, promote and increase sales of their products to maximise profits and generate continued shareholder value; these strategies are operationalised by the food industry through 3 spheres of action: political and legal; production, processing and design; and marketing and preference shaping.

The commercial determinants of dietary behavior and how they are operationalized (i.e., the three spheres of action) are conceptualized as being dynamic (changing over time), systemic (part of an interconnected web of actors), and targeting different levels in the system. The three spheres of action are underpinned by specific aims and target different levels in the system (see Table 1):

**TABLE 1:** Aims and levels of influence of the three spheres of action

Sphere of action	Aim	Levels of influence—examples
(1) Political and legal	Generate a business‐friendly regulatory and discursive environment	Macro—political and legal systems
		Meso—regulatory agencies, political parties, research and public health organizations, NGOs
		Micro—opinion leaders, government representatives, researchers, policymakers
(2) Production processing and design	Optimize processing and cost viability of their products	Macro—globalized market economies, emerging markets
		Meso—local organizations, manufacturing communities
(3) Marketing and preference shaping	Increase brand loyalty and enhance consumers' desire for their products	Meso—culture, social norms, consumer communities and groups, philanthropic communities, schools, sport venues
		Micro—individual consumers (e.g., children and adolescents)

Abbreviation: NGO, non‐governmental organization.

## 4 DISCUSSION

An integrative conceptualization of the commercial determinants of dietary behavior associated with obesity has been developed using principles from CIS.^27^ Findings suggest that dietary behavior associated with obesity is influenced by three spheres of action within corporations: *political and legal*; *production, processing, and design*; and *marketing and preference shaping*. These spheres of action build the structure of a system that influence and are influenced by different levels—the macro‐level (e.g., political and economic systems), meso‐level (e.g., research communities, sociocultural norms), and micro‐level (e.g., consumers). The *political and legal* sphere of action was the most frequently mentioned, followed by *marketing and preference shaping* and *production, processing, and design*. This highlights that research has mainly focused on how and what strategies have been developed to influence and shape the regulatory environment through the *political and legal* sphere of action. Within this sphere, the most reported corporate strategies were *influencing policymaking processes* through lobbying; *influencing governance of food production, trade, and investment* by taking advantage of neoliberal economic policies; and *framing the evidence and debate* by shaping the narrative of health and disease. In contrast, *marketing and preference shaping* was the most reported sphere of action in children/adolescence, which is a more proximal influence than *political and legal* sphere of action. This result aligns with the findings from Kelly et al.^104^ which proposes a conceptual pathway of effects of how marketing ultimately influences individual‐level weight outcomes. Although this review does not propose a logical sequence of effects linking marketing and preference shaping to weight status, the presented results enlist the specific practices food industry employs (i.e., advertising, sponsorship, sale promotions) that increase brand loyalty and enhance consumers' desire for their products which penetrates cultural and social norms as proposed by Cairns.^13^

Four articles were found to have potential CoIs,^33^, ^34^, ^35^, ^36^ but only one article did not provide evidence of any steps taken to mitigate against potential CoIs.^34^ This study gave a strong positive view on the food industry being part of the solution to obesity by focusing only on physical activity and undermining the role of processed food intake on obesity.

The current results are aligned to other frameworks found in the literature which focus on governance structures and corporate political activity of the food industry, with respect to public health.^15^, ^56^, ^65^, ^105^, ^106^ However, this review identified two additional spheres of corporation action, namely, *production, processing and design,* and *marketing and preference shaping,* which have different aims and mechanisms of action than the ones for corporate political activity.^107^ Two previous impact assessment frameworks^73^, ^108^ have highlighted that health impacts resulting from the actions of transnational corporations should be assessed not only according to their political practices, but also according to their business strategies. This review includes specific business and marketing practices from the food and beverage industry which are crucial in understanding the strategies employed to influence the food environment, dietary behavior, and obesity. Focusing only on corporate political activities would overshadow other important drivers and miss an opportunity to unpack the different levels of impact beyond influencing a business‐friendly regulatory environment.

There was overlap between some themes and subthemes in the proposed framework showing that some strategies and practices could also be categorized as part of other spheres of action. For example, CSR has been theorized to be part of corporate political activities to advance corporations' interests in terms of regulation ^65^; however, CSR can also be seen as part of a marketing strategy to raise brand awareness in targeted populations.^68^ Equally, product reformulation is part of the *production, processing, and design* sphere of action, but reformulation can also be used to shape narrative and debate on health and disease. Identifying these overlapping themes enables evaluating factors with an impact on multiple and different parts of the food system that influence dietary behavior and obesity.

While this article focused on the determinants of overnutrition (i.e., obesity), it is important to acknowledge that the economic causes of under and overnutrition could have a common structural basis, potentially driven by large corporations in demand for both, cheap labor and new consumers.

A major strength of this review is that it provides an integrative definition to describe the commercial determinants of dietary behavior and obesity and offers a conceptual framework to systematically study and identify how food industry's strategies and practices are operationalized to shape and influence dietary behaviors, and obesity at population level. The provided framework should be considered a conceptual starting point for future research and intervention development. In addition, this review puts commercial influences “back in the picture” by focusing explicitly and systematically on the commercial determinants of obesity, as suggested by Maani et al.^23^

A limitation of this study is that gray literature is not included. Additionally, inclusion criteria restricted articles to those written in English and Spanish. The search strategy retrieved articles that included the concept of commercial/corporate determinants, and therefore, articles using different terminology may have been excluded. Physical activity‐related literature was purposively left out since the focus of this review was on dietary behavior but might have revealed additional determinants.

The findings in this review highlight the *political and legal* and the *marketing and preference shaping* spheres of action as most frequently mentioned in the overall framework. However, this may reflect other relevant parts of the corporate sphere (*production, processing, and design*) being under‐theorized in the literature included in this review, potentially because of the distal effect these activities have on health outcomes.

The frequency of mention of a themes or subthemes was not assessed as an indicative of a sphere of action or strategy's importance or the size of its impact in the overall food system. It does not necessarily reflect the strength of the evidence but may reflect the attractiveness of the topic for researchers and funding, the different specialized fields, or difficulties in accessing data.

Data analysis and thus the definition and framework proposed incorporated expert‐opinion pieces and arguments supported by research data. A limitation of this approach is that the veracity of statements in commentary pieces could not be tested. However, expert‐opinion pieces can provide valuable insight into issues of broad concern in global health, particularly those concerning policy issues.^109^ Nonetheless, the sections in the framework that are dominated by opinion‐based arguments should be tested empirically to make this framework fully evidence‐based.

Future work should further explore the identified strategies and develop an in‐depth understanding of the mechanisms in the proposed framework by testing the level of impact of each strategy and translate and use these finding in the design of interventions. From a systems‐thinking perspective, this framework can be used to start exploring feedback loops, facilitate identifying and monitoring how the food system and dietary behavior patterns adapt over time, and anticipate industry reactions to regulation measures. Additionally, this framework can be used to highlight what is included and what has been left out in research or policymaking efforts.

## 5 CONCLUSIONS

This review provides a conceptual framework and an integrative definition of the commercial determinants of dietary behavior associated with obesity, specific to the food and beverage industry. The framework can enable a structured identification and systematic study of the impact of specific aspects of commercial strategies on the food environment, eating behavior, and obesity. It has the potential to be used in practice, policy, and research to identify levers for change in obesity prevention strategies, guide the development of health policies, and increase opportunities for primary prevention by anticipating industry responses and by discouraging corporate practices that harm health.

## CONFLICT OF INTERESTS

The authors have no competing interests to declare.

## AUTHORS CONTRIBUTIONS

Yanaina Chavez‐Ugalde, Russell Jago, Zoi Toumpakari, and Frank De Vocht conceived of the study. Yanaina Chavez‐Ugalde designed the study, conducted the search strategy, data collection, initial screening, and data analysis. Paige Hulls double screened titles and abstracts. Zoi Toumpakari did double coding in a proportion of the included studies to develop the definition and framework. Russell Jago, Zoi Toumpakari, and Frank De Vocht contributed to the methodological development of this study. All authors contributed to the data interpretation. Yanaina Chavez‐Ugalde drafted the manuscript, and all authors read, contributed, and revised the manuscript prior to publication.

## Supporting information

Supporting Information 1

## Footnotes

^1^
Note: For example: corn by‐products include flour, starch, oil, high fructose corn syrup, ethanol, livestock feed; soybean by‐products include: soybean hulls, meal, flour, oil, lecithin, livestock feed.
