# Supplementary material for: Conceptualizing the commercial determinants of dietary behaviors associated with obesity: A systematic review using principles from critical interpretative synthesis
Source: Obes Sci Pract. 2021 Apr 5;7(4):473–86. doi: 10.1002/osp4.507 (PMC8346378; doi:10.1002/osp4.507)
Supplement: Supplementary file 1 — Supporting Information 1 [file OSP4-7-473-s001.docx]

**Online supporting information**

**Article title:** Conceptualising the commercial determinants of dietary behaviours associated with obesity: a systematic review using principles from critical interpretative synthesis

**Authors**: Yanaina *Chavez-Ugalde^1,2^ Russell Jago ^1,3, 6^, Zoi Toumpakari ^3^, Matt Egan ^1,4^, Steven Cummins ^1,4^, Martin White^1,5^, Paige Hulls^2^, Frank De Vocht^1,2,6^*

^1^ NIHR School for Public Health Research

^2^ Population Health Sciences, Bristol Medical School, University of Bristol, UK

^3^ Centre for Exercise, Nutrition and Health Sciences, School for Policy Studies, University of Bristol, Bristol, UK

^4^ Department of Public Health, Environments and Society, London School of Hygiene and Tropical Medicine, London, UK

^5^ MRC Epidemiology Unit and Centre for Diet & Activity Research, University of Cambridge, UK

^6^ NIHR Applied Research Collaboration West (NIHR ARC West)

Contents

[1. Search strategy 2](#_Toc60581501)

[Table S1.1. Database and reference search with results 2](#_Toc60581502)

[Table S1.2. Search strategy key terms 2](#_Toc60581503)

[Table S1.3. Example of results in Medline database 2](#_Toc60581504)

[2. Table S2. Descriptive table of the 81 articles included 4](#_Toc60581505)

[3. Table S3. Detail on conflicts of interest based on Cullerton et al. (2019) Principles for preventing or managing CoI 10](#_Toc60581506)

[4. Table S4. Extracted quotes for definitions and mechanisms of the 81 articles included 18](#_Toc60581507)

[5. Table S5. Data-driven themes, subthemes and mechanisms 37](#_Toc60581508)

[6. Table S6. Prisma 2009 Checklist 42](#_Toc60581509)

#

# 1. Search strategy

## Table S1.1. Database and reference search with results

| **Database** | **Number of references retrieved** |
| --- | --- |
| Medline OvidSP | 2,987 |
| Pubmed | 503 |
| Web of science – Core Collection | 2,682 |
| Scopus | 3,117 |
|  | Total of 9,289 |
| Articles found through references | 35 |
|  | Total articles = 9,324 |
| Duplicates | 4,485 |
|  | Records after duplicates removed = 4,839 |

## Table S1.2. Search strategy key terms

| **Determinants** | **Industry** | **Dietary behaviour** | **Health outcome** |
| --- | --- | --- | --- |
| Commercial | Big food | Diet* behav* | Obesity |
| Commercial determinant* | Food | Food choice* | Overweight |
| Corporate | Drink | Dietary intake | Health |
| Corporate determinant* | Food industr* | Nutrition* |  |
|  |  | Eating behav* |  |

* possible endings of some words

[(commercial OR corporate).mp AND (determinant*).mp)] AND [(big food OR food OR drink).mp AND (industry*.mp)] AND [(diet* behav* OR food choice* OR dietary intake OR nutrition* OR eating behave*).mp] OR (obes* OR overweight OR health).mp]

## Table S1.3. Example of results in Medline database

- Medline (n=2,987)

1. "commercial".mp. (161516)
2. "commercial determinant*".mp. (32)
3. "corporate".mp. (7035)

4. 1 or 2 or 3 (168331)

5. "food".mp. (560734)

6. "food industr*".mp. (13085)

7. "big food".mp. (38)

8. "drink*".mp. (183819)

9. 5 or 6 or 7 or 8 (724057)

10. "obesity".mp. (305159)

11. "overweight".mp. (69194)

12. "health".mp. (2747912)

13. 10 or 11 or 12 (2987868)

14. "Diet* behav*".mp. (3513)

15. "Food choice*".mp. (4398)

16. "Dietary intake".mp. (23297)

17. "Nutrition*".mp. (368310)

18. "Eating behav*".mp. (9522)

19. 14 or 15 or 16 or 17 or 18 (393435)

20. 4 and 9 and 13 and 19 (804)

21. 13 or 19 (3252438)

22. 4 and 9 and 21 (2987)

## 2. Table S2. Descriptive table of the 81 articles included

| Author (year) | Publication type | Country / Region | Income level | Field of study | Population age group | Specific ages | Adolescents (10-19) | Health outcome | Conflicts of interest |
| --- | --- | --- | --- | --- | --- | --- | --- | --- | --- |
| Aaron & Siegel (2017) | Research article | USA | High | Public health | Not specified | Not specified | Not specified | Obesity | No risk |
| Anaf et al. (2017) | Research article | Australia | High | Public health | Children adolescents and adults | 0-9; 10-19; 20-34 | Yes | Obesity | No risk |
| Astrup et al. (2006) | Review article | USA and UK | High | Public health | Children and adolescents | Not specified | Not specified | Obesity | Potential conflict, but took measures to manage risk |
| Baker et al. (2020) | Research article | General | High, upper-middle, lower-middle and low | Nutrition | Children | 0-3 | No | Diet related NCDs | No risk |
| Barlow et al. (2018) | Perspective | General | High, upper-middle, lower-middle and low | Public health | Not specified | Not specified | Not specified | Obesity | No risk |
| Battams & Townsend (2019) | Research article | Switzerland, Australia, and Malaysia | High, upper-middle | Health policy | Not specified | Not specified | Not specified | Diet related NCDs | No risk |
| Biglan (2011) | Review article | USA | High | Public health | Children adolescents and adults | <18; 18-64; 65+ | Yes | Obesity | No risk |
| Brinsden & Lang (2015) | Commentary | General | High, upper-middle, lower-middle and low | Public health | Not specified | Not specified | Not specified | Obesity | No risk |
| Brownell & Warner (2009) | Review article | USA | High | Public health | Not specified | Not specified | Not specified | Obesity | No risk |
| Bump (2018) | Review article | General | High, upper-middle, lower-middle and low | Public health | Not specified | Not specified | Not specified | Obesity | No risk |
| Buse, Tanaka & Hawkes (2017) | Review article | General | High, upper-middle, lower-middle and low | Public health | Not specified | Not specified | Not specified | Diet related NCDs | No risk |
| Canella et al. (2015) | Perspective | Brazil | Upper- middle | Public health | Not specified | Not specified | Not specified | Obesity | No risk |
| Cannon (2004) | Review article | USA | High | Public health | Not specified | Not specified | Not specified | Diet related NCDs | No risk |
| Caraher & Cowburn (2015) | Commentary | Denmark | High | Health policy | Not specified | Not specified | Not specified | Dietary behaviour | No risk |
| Clapp & Scrinis (2017) | Perspective | General | High, upper-middle, lower-middle and low | Public health | Not specified | Not specified | Not specified | Diet related NCDs | No risk |
| Collin et al. (2017) | Research article | General | High, upper-middle, lower-middle and low | Health policy | Not specified | Not specified | Not specified | Diet related NCDs | No risk |
| de Lacy-Vawdon & Livingstone (2020) | Review article | General | High, upper-middle, lower-middle and low | Public health | Children adolescents and adults | Not specified | Not specified | Health | No risk |
| Delobelle et al. (2016) | Review article | South Africa | Upper- middle | Health policy | Not specified | Not specified | Not specified | Diet related NCDs | No risk |
| Dixon, Sindall & Banwell (2004) | Review article | Australia | High | Public health | Not specified | Not specified | Not specified | Dietary behaviour | No risk |
| Dorfman et al. (2012) | Review article | USA and UK | High | Health policy | Adolescents and adults | 11 to 31 years | Yes | Obesity | No risk |
| Douglas et al. (2018) | Research article | England | High | Public health | Not specified | Not specified | Not specified | Obesity | No risk |
| Eastmure et al. (2020) | Review article | General | High | Business | Not specified | Not specified | Not specified | Health | No risk |
| Fabbri et al. (2018) | Research article | Australia, Great Britain, New Zealand, USA and Canada | High | Public health | Not specified | Not specified | Not specified | Obesity | No risk |
| Fooks et al. (2019) | Research article | South Africa | Upper- middle | Health policy | Not specified | Not specified | Not specified | Diet related NCDs | No risk |
| Franz & Kickbusch (2018) | Commentary | Europe | High, upper- and lower-middle | Public health | Not specified | Not specified | Not specified | Diet related NCDs | No risk |
| Freudenberg (2012) | Perspective | USA | High | Public health | Not specified | Not specified | Not specified | Obesity | No risk |
| Freudenberg (2005) | Review article | USA | High | Health education | Not specified | Not specified | Not specified | Obesity | No risk |
| Freudenberg (2018) | Commentary | General | High, upper-middle, lower-middle and low | Public health | Not specified | Not specified | Not specified | Diet related NCDs | No risk |
| Freudenberg & Galea (2008) | Review article | USA | High | Public health | Not specified | Not specified | Not specified | Obesity | No risk |
| Freudenberg & Galea (2008) | Review article | USA | High | Public health | Not specified | Not specified | Not specified | Diet related NCDs | No risk |
| Green (2019) | Commentary | UK | High | Public health | Not specified | Not specified | Not specified | Health | No risk |
| Greenhalgh (2016) | Review article | China | Upper- middle | Anthropology | Not specified | Not specified | Not specified | Obesity | No risk |
| Grotz (2006) | Review article | USA | High | Nutrition | Children and adolescents | <12 years | Yes | Obesity | Potential conflict |
| Handsley & Reeve (2018) | Review article | Australia | High | Law | Children and adolescents | <18 years | Yes | Obesity | No risk |
| Henriques et al. (2018) | Research article | Brazil | Upper- middle | Health policy | Children and adolescents | Not specified | Yes | Obesity | No risk |
| Herrick (2009) | Research article | USA and UK | High | Sociology | Not specified | Not specified | Not specified | Obesity | No risk |
| Ireland (2019) | Commentary | General | High, upper-middle, lower-middle and low | Health policy | Not specified | Not specified | Not specified | Diet related NCDs | No risk |
| Ireland (2019) | Review article | Australia, New Zealand, Turkey, USA | High and upper- middle | Public health | Not specified | Not specified | Not specified | Diet related NCDs | No risk |
| Jahiel (2008) | Research article | USA | High | Public health | Not specified | Not specified | Not specified | Obesity | No risk |
| Jaichuen et al. (2018) | Research article | Thailand | Upper- middle | Health policy | Not specified | Not specified | Not specified | Diet related NCDs | No risk |
| Jane & Gibson (2017) | Research article | Great Britain | High | Public health | Children and adolescents | <15 years | Yes | Diet related NCDs | No risk |
| Kickbusch (2016) | Commentary | General | High, upper-middle, lower-middle and low | Public health | Not specified | Not specified | Not specified | Health | No risk |
| Knai et al. (2018) | Research article | General | High, upper-middle, lower-middle and low | Public health | Not specified | Not specified | Not specified | Diet related NCDs | No risk |
| Kraak, Kumanyika & Story (2009) | Review article | General | High, upper-middle, lower-middle and low | Public health | Children and adolescents | Not specified | Yes | Obesity | No risk |
| Kraak et al. (2012) | Review article | General | High, upper-middle, lower-middle and low | Public health | Children adolescents and adults | Not specified | Yes | Diet related NCDs | Potential conflict, but took measures to manage risk |
| Lacy-Nichols et al. (2020) | Research article | Australia | High | Health policy | Not specified | Not specified | Not specified | Obesity | No risk |
| Lauber et al. (2020) | Research article | General | High, upper-middle, lower-middle and low | Health policy | Not specified | Not specified | Not specified | Diet related NCDs | No risk |
| Lencucha et al. (2018) | Perspective | General | High, upper-middle, lower-middle and low | Health policy | Not specified | Not specified | Not specified | Diet related NCDs | No risk |
| Lencucha & Thow (2019) | Perspective | General | High, upper-middle, lower-middle and low | Health policy | Not specified | Not specified | Not specified | Diet related NCDs | No risk |
| Leon & Ken (2019) | Perspective | USA and China | High and upper- middle | Law | Not specified | Not specified | Not specified | Diet related NCDs | No risk |
| Lobstein et al. (2015) | Review article | General^1^ | High, upper-middle, lower-middle and low | Obesity | Children and adolescents | Not specified | Yes | Obesity | No risk |
| Maani-Hessari et al. (2019) | Research article | USA | High | Public health | Not specified | Not specified | Not specified | Obesity | No risk |
| Madureira-Lima (2019) | Research article | General | High, upper-middle, lower-middle and low | Public health | Not specified | Not specified | Not specified | Diet related NCDs | No risk |
| McKee & Stuckler (2018) | Commentary | General | High, upper-middle, lower-middle and low | Public health | Not specified | Not specified | Not specified | Obesity | No risk |
| McKee, Steele & Stuckler (2019) | Commentary | China | Upper- middle | Public health | Not specified | Not specified | Not specified | Obesity | No risk |
| Mialon (2020) | Review article | General | High, upper-middle, lower-middle and low | Public health | Children adolescents and adults | Not specified | Not specified | Health | No risk |
| Mialon et al. (2019) | Research article | France | High | Public health | Not specified | Not specified | Not specified | Diet related NCDs | No risk |
| Mialon & Mialon (2018) | Research article | France | High | Public health | Not specified | Not specified | Not specified | Diet related NCDs | No risk |
| Mialon, Swinburn & Sacks (2015) | Review article | General | High, upper-middle, lower-middle and low | Public health | Not specified | Not specified | Not specified | Diet related NCDs | No risk |
| Millar (2013) | Commentary | Canada | High | Public health | Not specified | Not specified | Not specified | Diet related NCDs | No risk |
| Miranda et al. (2019) | Review article | General^2^ | Middle (upper and lower- middle) | Public health | Children adolescents and adults | Not specified | Not specified | Diet related NCDs | No risk |
| Molnar (2005) | Perspective | USA | High | Health policy | Children and adolescents | 6-19 years | Yes | Obesity | No risk |
| Moodie et al. (2013) | Review article | General | High, upper-middle, lower-middle and low | Public health | Not specified | Not specified | Not specified | Diet related NCDs | Potential conflict, but took measures to manage risk |
| Moodie et al. (2006) | Commentary | Canada, Europe, USA, Australia, Norway, Sweden | High | Obesity | Children and adolescents | Not specified | Not specified | Obesity | No risk |
| Nestle (2000) | Perspective | USA | High | Public health | Children and adolescents | 6-19 years | Yes | Obesity | No risk |
| Olstad & Raine (2013) | Commentary | Canada | High | Public health | Children and adolescents | Not specified | Not specified | Dietary behaviour | No risk |
| Panjwani & Caraher (2014) | Research article | UK | High | Health policy | Not specified | Not specified | Not specified | Dietary behaviour | No risk |
| Powell (2016) | Research article | New Zealand | High | Health education | Children and adolescents | 5-13 years | Yes | Obesity | No risk |
| Raine (2013) | Review article | UK | High | Public health | Children and adolescents | 6-12 years | Yes | Obesity | No risk |
| Schram, Labonté & Sanders (2013) | Research article | Sub-Saharan Africa ^3^ | Upper and lower- middle | Public health | Not specified | Not specified | Not specified | Diet related NCDs | No risk |
| Schrempf (2014) | Research article | General | High, upper-middle, lower-middle and low | Business | Not specified | Not specified | Not specified | Obesity | No risk |
| Smith, Buse & Gordon (2016) | Commentary | General | High, upper-middle, lower-middle and low | Public health | Not specified | Not specified | Not specified | Dietary behaviour | No risk |
| Sonntag et al. (2015) | Review article | General ^4^ | High | Public health | Children and adolescents | 3-11 years | Yes | Obesity | No risk |
| Stuckler & Nestle (2012) | Perspective | General | High, upper-middle, lower-middle and low | Public health | Not specified | Not specified | Not specified | Obesity | No risk |
| Stuckler et al. (2012) | Research article | General | High, upper-middle, lower-middle and low | Health policy | Not specified | Not specified | Not specified | Diet related NCDs | No risk |
| Swinburn (2008) | Commentary | General^5^ | High, upper-middle, lower-middle and low | Health policy | Not specified | Not specified | Not specified | Obesity | No risk |
| Swinburn et al. (2019) | Lancet commission | General | High, upper-middle, lower-middle and low | Public health | Not specified | Not specified | Not specified | Diet related NCDs | No risk |
| Thorn (2018) | Commentary | Australia | High | Public health | Not specified | Not specified | Not specified | Obesity | No risk |
| Weishaar et al. (2016) | Review article | USA and UK | High | Public health | Not specified | Not specified | Not specified | Diet related NCDs | No risk |
| Wells (2013) | Review article | General | High, upper-middle, lower-middle and low | Nutrition | Children and adolescents | <15 years | Yes | Obesity | No risk |
| West & Marteau (2013) | Commentary | General | High, upper-middle, lower-middle and low | Public health | Not specified | Not specified | Not specified | Health | No risk |
| Notes: Region with specific mention of countries:   \| ^1^ High (USA, Australia, Finland, Canada, NZ, Spain, UK, Saudi Arabia, Seychelles, Hong Kong)- upper middle (Mexico, Brazil, Iran, South Africa, China) \| \| --- \| \| ^2^ Low-and middle-income countries "LMIC"--> upper and lower- middle (Brazil, Chile, China, Ghana, Guatemala, India, Mexico, Peru, South Africa, Thailand, Uruguay, Vietnam) \| \| ^3^ Cameroon, Kenya, Nigeria, and South Africa \| \| ^4^ European countries, USA, Canada, Australia, and New Zealand \| \| ^5^Tonga and India \| | | | | | | | | |  |

# 3. Table S3. Detail on conflicts of interest based on Cullerton et al. (2019) Principles for preventing or managing CoI

| **Author (year)** | **Cullerton et al. (2019) Principles for preventing or managing CoI (Funding, Risk Assessment, Research Governance, Transparency, Publication)** | **Detail for potential conflict of interest** |
| --- | --- | --- |
| **Aaron & Siegel (2017)** | No conflict of interests declared, and potentially no risk |  |
| **Anaf et al. (2017)** | No conflict of interests declared, and potentially no risk |  |
| **Astrup et al. (2006)** | Potential conflict of interest regarding to the funding and risk assessment of the project since the funding came from a company who's objectives are related to increase "unhealthy food" consumption. However, measures were taken to manage the potential conflict of interest and there seems to be no influence on findings of the research. | "The work reported here was sponsored by Procter & Gamble, producer of Pringles brand potato chips. The contents of this publication do not necessarily reﬂect the views or policies of Procter & Gamble, though. The authors assume full responsibility for the ﬁnal text."  Authors took measures to prevent or manage conflict of interest through trying to limit the involvement of the funder in any aspects of the project [statement 1.10]; explicitly reporting the nature of funding received from food industry [statement 4.1]; included findings that were unfavourable to the funder [statement 5.2]; included a statement that results from the article do not necessarily reflect the views expressed by the funder [statement 5.3]. |
| **Baker et al. (2020)** | No conflict of interests declared, and potentially no risk |  |
| **Barlow et al. (2018)** | No conflict of interests declared, and potentially no risk |  |
| **Battams & Townsend (2019)** | No conflict of interests declared, and potentially no risk |  |
| **Biglan (2011)** | No conflict of interests declared, and potentially no risk |  |
| **Brinsden & Lang (2015)** | No conflict of interests declared, and potentially no risk |  |
| **Brownell & Warner (2009)** | No conflict of interests declared, and potentially no risk |  |
| **Bump (2018)** | No conflict of interests declared, and potentially no risk |  |
| **Buse, Tanaka & Hawkes (2017)** | No conflict of interests declared, and potentially no risk |  |
| **Canella et al. (2015)** | No conflict of interests declared, and potentially no risk |  |
| **Cannon (2004)** | No explicit declaration, but potentially no conflict of interest | Affiliations:  World Health Policy Forum, NY, USA  No connection to commercial entities |
| **Caraher & Cowburn (2015)** | No explicit declaration, but potentially no conflict of interest | Affiliations: Martin Caraher: Centre for Food Policy, Department of Sociology, City University London, UK  Gill Cowburn: British Heart Foundation Centre on Population Approaches for Non-Communicable Disease Prevention, Nuffield Department of Population Health, University of Oxford, UK  No connection to commercial entities |
| **Clapp & Scrinis (2017)** | No conflict of interests declared, and potentially no risk |  |
| **Collin et al. (2017)** | No conflict of interests declared, and potentially no risk |  |
| **de Lacy-Vawdon & Livingstone (2020)** | No conflict of interests declared, and potentially no risk | This research has not received any project specific funding. CdV is a PhD student supported by an Australian Government Research Training Program Scholarship, and a Monash Graduate Excellence Scholarship provided by Monash University. Previously, CdV has also received project funding from the Susan McKinnon Foundation, the Victorian Responsible Gambling Foundation, the Victorian Department of Health and Human Services, municipal councils in Victoria, Australia, MOVE Muscle, Bone & Joint Health, and Impact Investment Group. CdV has also worked for the Australian Gambling Research Centre at the Australian Institute of Family Studies, and received funding from the Australian Government Department of Social Services in this role. CL has received funding from the Victorian Responsible Gambling Foundation, the (former) Victorian Gambling Research Panel, and the South Australian Independent Gambling Authority (the funds for which were derived from hypothecation of gambling tax revenue to research purposes), from the Australian and New Zealand School of Government and the Foundation for Alcohol Research and Education, and from non-government organizations for research into multiple aspects of EGM gambling, including regulatory reform, existing harm minimization practices, and technical characteristics of gambling forms. He has received travel and co-operation grants from the Turkish Green Crescent Society, Alberta Problem Gambling Research Institute, the Finnish Institute for Public Health, the Finnish Alcohol Research Foundation, the Ontario Problem Gambling Research Committee, and the Problem Gambling Foundation of New Zealand. He was a Chief Investigator on an Australian Research Council project researching mechanisms of influence on government by the tobacco, alcohol and gambling industries. He has undertaken consultancy research for local governments and nongovernment organizations in Australia and the UK seeking to restrict or reduce the concentration of poker machines and gambling impacts, and was a member of the Australian government’s Ministerial Expert Advisory Group on Gambling in 2010–11, remuneration for which was paid to Monash University. |
| **Delobelle et al. (2016)** | No conflict of interests declared, and potentially no risk |  |
| **Dixon, Sindall & Banwell (2004)** | No conflict of interests declared, and potentially no risk |  |
| **Dorfman et al. (2012)** | No conflict of interests declared, and potentially no risk |  |
| **Douglas et al. (2018)** | No conflict of interests declared, and potentially no risk |  |
| **Eastmure et al. (2020)** | No conflict of interests declared, and potentially no risk |  |
| **Fabbri et al. (2018)** | No conflict of interests declared, and potentially no risk |  |
| **Fooks et al. (2019)** | No conflict of interests declared, and potentially no risk |  |
| **Franz & Kickbusch (2018)** | No conflict of interests declared, and potentially no risk |  |
| **Freudenberg (2012)** | No conflict of interests declared, and potentially no risk |  |
| **Freudenberg (2005)** | No conflict of interests declared, and potentially no risk |  |
| **Freudenberg (2018)** | No conflict of interests declared, and potentially no risk |  |
| **Freudenberg & Galea (2008)** | No conflict of interests declared, and potentially no risk |  |
| **Freudenberg & Galea (2008)** | No conflict of interests declared, and potentially no risk |  |
| **Green (2019)** | No explicit declaration, but potentially no conflict of interest | Affiliations:  School of Population & Environmental Health Sciences, King’s College London No connection to commercial entities |
| **Greenhalgh (2016)** | No conflict of interests declared, and potentially no risk |  |
| **Grotz (2006)** | Potential conflict of interest.  Author disclosed affiliation and employer details (directly employed by food industry). However, seems that no measures were taken to manage the potential conflict of interest.   The study only includes findings favourable to the financial supporters of the study and to the sponsors of the journal where it was published. There seems to be strong influence on findings of the research. | Dr. Grotz published this article while being Director of Product Safety at McNeil Nutritionals, LLC (producer of Lactaid, Splenda, Benecol), a **Johnson & Johnson** Company. Additionally, the publication was made on Nutrition Reviews Journal "Nutrition Reviews, published by the International Life Sciences Institute in partnership with Oxford University Press." ILSI-sponsored research is funded by grants from companies related to the food business.  The study gives a strong positive view on the food industry being part of the solution to obesity focusing on the physical activity, creating new products, self-regulation pledges on marketing, creating new collaborations and having fully industry funded conferences and symposia, and giving unrestricted grants to researchers, but undermining the role processed food intake.   The author was transparent in reporting employers' information, however, they did not view this as a potential conflict of interest nor did they include any statement establishing a clear definition of who had control over the preparation and approval of peer-reviewed manuscript [statement 5.3] nor the level of involvement of the employer in any aspect of the project [statement 1.10].   Overall, author did not take measures to prevent or manage conflict of interest with regards to 1) structure and governance of funding; 2) undertaking a risk assessment; 3) maintaining high standards of research governance, 4) ensuring high levels of transparency; 5) improving publication standards. |
| **Handsley & Reeve (2018)** | No explicit declaration, but potentially no conflict of interest | Affiliations: Elizabeth Handsley: Professor of Law, Flinders University Belinda Reeve: Senior Lecturer, University of Sydney Law School  No connection to commercial entities |
| **Henriques et al. (2018)** | No conflict of interests declared, and potentially no risk |  |
| **Herrick (2009)** | No conflict of interests declared, and potentially no risk |  |
| **Ireland (2019)** | No conflict of interests declared, and potentially no risk |  |
| **Ireland (2019)** | No conflict of interests declared, and potentially no risk |  |
| **Jahiel (2008)** | No conflict of interests declared, and potentially no risk |  |
| **Jaichuen et al. (2018)** | No conflict of interests declared, and potentially no risk |  |
| **Jane & Gibson (2017)** | No explicit declaration, but potentially no conflict of interest | Affiliations: B. Jane and K. Gibson: Faculty of Sport and Health, The University of St Mark & St John, Plymouth, UK  No connection to commercial entities |
| **Kickbusch (2016)** | No conflict of interests declared, and potentially no risk |  |
| **Knai et al. (2018)** | No conflict of interests declared, and potentially no risk |  |
| **Kraak, Kumanyika & Story (2009)** | No conflict of interests declared, and potentially no risk |  |
| **Kraak et al. (2012)** | Potential conflict of interest based on the place of employment of one of the authors. However, measures were taken to manage the potential conflict of interest and there seems to be no influence on findings of the research as it includes findings unfavourable to the financial supporters of the employee. | "P.B.H. is employed by Save the Children, which in the USA has received funding to support several programme areas Among these supporters are The Bill and Melinda Gates Foundation, General Mills Foundation, Kidfresh, Kraft Foods and Kraft Foundation, PepsiCo Foundation, Wal-Mart Foundation and the Wm. Wrigley Jr. Company Foundation. The views expressed here are those of the authors and not necessarily those of Save the Children Federation"  Authors took measures to prevent or manage conflict of interest through limiting the involvement of the funder in any aspects of the project [statement 1.10]; explicitly reporting the nature of funding received from food industry by the employer [statement 4.1]; included a statement of potential conflicts of interests from all authors [statement 5.1]; included findings that were unfavourable to the funder [statement 5.2]; included a statement that results from the article do not necessarily reflect the views expressed by the employer [statement 5.3]. |
| **Lacy-Nichols et al. (2020)** | No conflict of interests declared, and potentially no risk |  |
| **Lauber et al. (2020)** | No conflict of interests declared, and potentially no risk |  |
| **Lencucha et al. (2018)** | No conflict of interests declared, and potentially no risk |  |
| **Lencucha & Thow (2019)** | No conflict of interests declared, and potentially no risk |  |
| **Leon & Ken (2019)** | No explicit declaration, but potentially no conflict of interest | Affiliations: Kenneth Leon: Department of Latino & Caribbean Studies, Rutgers University, New Brunswick, NJ, USA Ivy Ken: Department of Sociology, George Washington University, Washington, DC, USA  No connection to commercial entities |
| **Lobstein et al. (2015)** | No conflict of interests declared, and potentially no risk |  |
| **Maani-Hessari et al. (2019)** | No conflict of interests declared, and potentially no risk |  |
| **Madureira-Lima (2019)** | No conflict of interests declared, and potentially no risk |  |
| **McKee & Stuckler (2018)** | No conflict of interests declared, and potentially no risk |  |
| **McKee, Steele & Stuckler (2019)** | No conflict of interests declared, and potentially no risk |  |
| **Mialon (2020)** | No conflict of interests declared, and potentially no risk |  |
| **Mialon et al. (2019)** | No conflict of interests declared, and potentially no risk |  |
| **Mialon & Mialon (2018)** | No conflict of interests declared, and potentially no risk |  |
| **Mialon, Swinburn & Sacks (2015)** | No conflict of interests declared, and potentially no risk |  |
| **Millar (2013)** | No conflict of interests declared, and potentially no risk |  |
| **Miranda et al. (2019)** | No conflict of interests declared, and potentially no risk |  |
| **Molnar (2005)** | No explicit declaration, but potentially no conflict of interest | Affiliations: Alex Molnar: Education Policy Studies Laboratory, College of Education, Arizona State University, USA  No connection to commercial entities |
| **Moodie et al. (2013)** | Potential conflict of interest regarding to the funding and risk assessment of the project since some of the authors, prior to this project, received travel or lecture fees or reinbursements from a company who's objectives are related to increase "unhealthy food" consumption. However, measures were taken to manage the potential conflict of interest and there seems to be no influence on findings of the research. | "BN is the Chair of the Australian Division of World Action on Salt and Health (2007–ongoing)...**He has received lecture fees, travel fees, or reimbursements from** (...) **PepsiCo** (2011) (...). He holds research support from the Australian Food and Grocery Council (2012), (...) He is **not employed by a commercial entity; has no equity ownership or stock options, patents, or royalties; and has no other ﬁnancial or non-ﬁnancial support that might be viewed as creating potential conﬂicts of interest**. NS is co-chair of the UK Government Responsibility Deal network, trustee of the Drinkaware Trust, member of (...) bodies with alcohol industry representation. NS is also a member of the Alcohol Health Alliance UK executive board, Royal College of Physicians’ alcohol committee, and is involved in other health NGOs including Alcohol Concern. None of these commitments included ﬁnancial gain but some have reimbursed travelling expenses."   Authors took measures to prevent or manage conflict of interest since although having accepted reimbursements from food industry in the past, researchers had no commercial interest or financial gain [statement1.6]; explicitly reporting the nature of funding received from food industry [statement 4.1]; included findings that were unfavourable to food industry [statement 5.2]. |
| **Moodie et al. (2006)** | No explicit declaration, but potentially no conflict of interest | Affiliations: Rob Moodie: Victorian Health Promotion Foundation, Victoria, Australia  Boyd Swinburn: School of Exercise and Nutrition Sciences, Deakin University, Melbourne, Australia  Jeff Richardson: Centre for Health Economics, Faculty of Business and Economics, Monash University, Melbourne, Australia, Bertino Somaini: Health Promotion Switzerland, Bern, Switzerland  No connection to commercial entities |
| **Nestle (2000)** | No conflict of interests declared, and potentially no risk |  |
| **Olstad & Raine (2013)** | No conflict of interests declared, and potentially no risk |  |
| **Panjwani & Caraher (2014)** | No explicit declaration, but potentially no conflict of interest | Clare Panjwani and Martin Caraher: Centre for Food Policy, Department of Sociology, City University London, UK  No connection to commercial entities |
| **Powell (2016)** | No conflict of interests declared, and potentially no risk |  |
| **Raine (2013)** | No explicit declaration, but potentially no conflict of interest | Affiliations: Gary Raine: Leeds Metropolitan University, Institute for Health & Wellbeing, Faculty of Health & Social Sciences, Leeds, UK   No connection to commercial entities |
| **Schram, Labonté & Sanders (2013)** | No conflict of interests declared, and potentially no risk |  |
| **Schrempf (2014)** | No conflict of interests declared, and potentially no risk |  |
| **Smith, Buse & Gordon (2016)** | No conflict of interests declared, and potentially no risk |  |
| **Sonntag et al. (2015)** | No conflict of interests declared, and potentially no risk |  |
| **Stuckler & Nestle (2012)** | No conflict of interests declared, and potentially no risk |  |
| **Stuckler et al. (2012)** | No conflict of interests declared, and potentially no risk |  |
| **Swinburn (2008)** | No conflict of interests declared, and potentially no risk |  |
| **Swinburn et al. (2019)** | No conflict of interests declared, and potentially no risk |  |
| **Thorn (2018)** | No conflict of interests declared, and potentially no risk |  |
| **Weishaar et al. (2016)** | No conflict of interests declared, and potentially no risk |  |
| **Wells (2013)** | No conflict of interests declared, and potentially no risk |  |
| **West & Marteau (2013)** | No conflict of interests declared, and potentially no risk |  |

# 4. Table S4. Extracted quotes for definitions and mechanisms of the 81 articles included

| **Author (year)** | **Definition for the commercial/corporate determinants** |
| --- | --- |
| Aaron & Siegel (2017) | "Sponsorship (of national health organisations by soda companies) are likely to serve marketing functions, such as to dampen health groups’ support of legislation that would reduce soda consumption and improve soda companies’ public image" |
| Anaf et al. (2017) | "The practices of transnational corporations (TNCs) affect population health through production methods, shaping social determinants of health, or influencing the regulatory structures governing their activities "  **Political** practices. "McDonald’s Australia’s ... engages **lobbyists**, ... to ensure the **least restrictive regulatory environments**. • McDonald’s engages in strategic industry alliances that can help influence regulatory oversight and promote corporate interests over health and wellbeing. This includes the integrated and creative marketing directed to children and young people. "  **Business** practices." • McDonald’s taxation strategies undermine governments’ ability to fund health and welfare policies including funding for corporate monitoring and regulation. • new outlet expansion * Taxation minimisation strategies - taxation strategies that bolster corporate profitability. Under international taxation legal structures, transfer pricing between two of the same companies allows for distortions in the price of trade, or transfer ‘**mispricing**’; and for minimising taxation through reporting profits in tax havens"  **Products:** "Products • Many of McDonald’s food products are ultra-processed, high in kilojoules, fats, sugar and sodium. These can lead to obesity. McDonald’s online ordering, drive through outlets, and home delivery all provide ease of access to unhealthy products."  **Marketing** • McDonald’s engages high profile media support which may help strengthen **integrated marketing** to children. This promotes brand choices linked to unhealthy food and childhood obesity. • A range of **sponsorships** promotes purchasing practices and creates a Halo effect - deflects criticism from health damaging products (mascots, bolstering charitable purposes)  **Equity impacts**. " However, there is **externalisation of costs** to the public from profit shifting, tax havens, and service fees paid back to USA headquarters. • The health costs of non-communicable diseases and environmental impacts from McDonald’s operations are externalised to the community. -- Increased housing prices due to having a McDonalds near. |
| Astrup et al. (2006) | "Commerce is considered a strong force in the shaping of the obesity epidemic. It produces a never-ending stream of messages urging and seducing us to eat (and drink). Particularly criticized is the marketing of products with a low nutritional quality, aimed at kids. Commerce has made food, also the less wholesome varieties, available everywhere at any time and at low costs. A part of the commercial seduction process, which many indicate as contributing to obesity, is the offering of ever-larger portions and multiple packaging... great offer, the intensive marketing and the price of calorie-rich products" |
| Baker et al. (2020) | "Infant and young child feeding is often portrayed as an **individual behaviour, as a matter of free parental choice**, and the responsibility of mothers and parents alone. Our findings present a counter-view – feeding decisions are powerfully shaped by **transformations underway in first-food systems**, associated with the **globalization** of the baby food industry and its **marketing practices,** processes of medicalisation and limited support for breastfeeding among healthcare professionals, and the shift of labour and production out of the home, in the context of weak or absent maternity protection and breastfeeding support.(...) Actions are also needed to curtail interactions between baby f**ood companies and health professionals,** including conflicts of interest." "The **liberalization of trade in retail** services has spurred the ‘supermarketization’ of developing countries since the late 1990s, creating new opportunities to market baby food products to urban consumers with rising incomes. Supermarketisation links with a product **differentiation and line extension marketing strategy,** because by expanding product ranges in these ways, companies can acquire more **shelf space in supermarkets** and pharmacies, further promoting product visibility and sales. Online and in-store retailers often violate The Code, by **price-discounting or running in-store promotions** to move stock." |
| Barlow et al. (2018) | "Advance (Coca-Cola's) intrests by funding a scientific organisation (GEBN). Coca-Cola intended to use the GEBN to: (i) reframe obesity as a matter of addressing ‘energy balance’; (ii) portray the GEBN as an ‘honest broker’ in the obesity debate; (iii) promote obesity reduction strategies that are commensurate with Coca-Cola’s interests via an extensive advocacy campaign."... ‘promote practices that are effective in terms of both policy and profit (emphasis added)’. |
| Battams & Townsend (2019) | "The study suggested powerful ideologies embedded within governments and international institutions create a reluctance to challenge trade interests and the health impacts of trade. There also appeared to be considerable power within governments’ geo-political alliances based upon **trade interests, and industry coalitions** targeting countries with less regulatory resistance. This suggested some support for ‘advocacy coalitions’ operating based upon trade and geopolitical interests."  "we found that competing frames within trade and health sectors are reflective of very different core objectives; on the one hand, to expand exports and market liberalisation, and, on the other, to promote health and control NCD risk factors. **Power asymmetries** that favour trade sectors at the national and international level enable trade actors, industry advocates and politicians to control the extent to which health concerns do or do not get onto the agenda. (...) Underlying these policy domains is the ‘deep core’ of **neoliberalism**, which promotes individual responsibility, industry actors’ direct involvement in policymaking and a reduced role for the state in regulation. The dominance of neoliberal ideas in contemporary governance promotes the privileging of export interests for economic development, resistance to regulation and ‘individual responsibility’ frames for NCD risk factors." |
| Biglan (2011) | Michael Pollan (2007) recently pointed out that "...the low cost of high calorie foods with little nutritional value is due, in part, to federal **subsidies** for production of corn and soybeans. It is likely— and surely worthy of evaluation—that reducing these subsidies would reduce the consumption of unhealthy foods. "A negative corporate **externality** is the harm that the business transaction of a corporation does to a third party (Organisation for Economic Cooperation and Development 2008). "  " some corporate practices are risk factors for psychological, behavioral, and physical ill health... Food marketing and **production practices** are important risk factors for obesity (...) food and beverage marketers’ and fast-food restaurants’ ongoing marketing and sales promotion efforts, such as value pricing, psychological pricing, quantity discounts, and combo deals, which undermine portion control and healthy food choices" |
| Brinsden & Lang (2015) | " Corporate responsibility (CR) is a poor substitute for market reframing, but CR has been offered as the route to improved diet action, with companies promising product reformulation as the key to obesity… Changing product recipes may be good brand protection but has little population dietary impact, and is no compensation for vast marketing budgets. Some sections of the food industry are ideologically opposed to public health intervention, arguing that it demeans individual choice. They favour a technical approach to nutrition to justify the products that they produce and sell. ... vast **marketing** and **distribution** budgets of ‘Big Food’ mean the relationship between producer and consumer is unequal"  ""solutions to **poverty** and malnutrition have been largely penetrated by a prevailing market ethos which critics see as favouring deference to multi-national corporations, foreign direct investment and a processed food paradigm" |
| Brownell & Warner (2009) | " The food industry appears to have a strategy as well, repeatedly carried to the public by spokespersons from food companies, trade associations, and their political allies… : Focus on **personal responsibility** as the cause of the nation's un healthy diet. **Raise fears** that government action usurps **personal freedom**. **Vilify critics** with totalitarian language, characterizing them as the food police, leaders of a **nanny state**, and even "food fascists," and accuse them of desiring to **strip people of their civil liberties**. C**riticize studies that hurt industry** as "junk science." **Emphasize physical activity over diet**. State there are **no good or bad foods**; hence no food or food type (soft drinks, fast foods, etc.) should be targeted for change. **Plant doubt** when concerns are raised about the industry."  "While working to promote healthy eating, the USDA at the same time has as its main objective the promotion of American agriculture (selling more food), so one goal typically prevails over the other when the two conflict. There is a long history of USDA leaders and leaders of other agencies being recruited from food and agriculture industries and then returning to businesses like lobbying firms when their government service ends." |
| Bump (2018) | "Very different views on governance came from studies focusing on obesity, where primary themes include the globalisation of food, the products and marketing of multinational corporations (MNCs) and individual lifestyle choices. Globalisation has changed the landscape of food and nutrition by concentrating power in large firms, changing consumer taste through marketing, and limiting the effectiveness of traditional governance mechanisms. "  "Obesity problems are characterised by an insidious dynamic... Accountability and participation can be strong, but may be corrupted by marketing and other influences on citizen perception. The demands expressed by citizens may be accurately—and harmfully—expressed in unhealthy foods, habits and environments. This shifts the intervention area from governance structures per se to the inputs—the things that citizens want—and can be characterised as a **contest for perceptions** about good choices in food, food policy and related issues of regulation, tax and trade. " |
| Buse, Tanaka & Hawkes (2017) | "CDoH are the driving risks of ill-health (...) profit-driven epidemics”  "Some critics warn that any partnership creates benefit for industry (indeed industry must benefit for sustained engagement) – but see no clear, established or legitimate mechanism through which public health would be protected." |
| Canella et al. (2015) | " industries have made use of their relationships with scientific institutions, associations, and medical professionals to leverage the marketing of their products through investments in research, honoraria, advisory services, or donations, or by **financing scientific events with sponsorships.** In addition, these industries have employed many marketing strategies to showcase apparent public health benefits while aiming, in fact, to serve mainly their own economic interests. "  "Despite evidence of social determinants of health and disease that are often directly related to a lack of regulation of the food and beverage industries, these companies underscore the individual’s “freedom of choice” in order to obstruct legislative initiatives aimed at regulating the **production, marketing**, and **advertising** of their products." |
| Cannon (2004) | "The nature and quality of national, international and global food systems change, and with them, patterns of health and disease; and how and why the powers dominant in the world at any given time have manipulated food systems in order to extend their dominion and to become more rich and more powerful." |
| Caraher & Cowburn (2015) | "Three policy streams of ‘problem’, ‘politics’ and ‘context’ need to overlap for policy to occur. The context and problem can, of course, be reformulated by business interests. A well-used approach for alcohol, tobacco and, more recently, food-related corporate interests is to shift the focus away from health. This involves **reframing** a fat or soft drinks tax as an issue of **consumer rights** and a debate over the role of the state in ‘**nannying**’ or restricting people's choices (...) Building support for policies is never just a matter of evidence. In public health and preventive medicine there is a long history of interventionist public health policy."  " The new and **powerful influences** are the **corporate interests** and the influence of **neo-liberal** economics above and beyond health. The **corporate capture of public health** is epitomised by government's eagerness to enter into voluntary agreements, which place the views of industry above those evidence-based findings that prioritize public health." |
| Clapp & Scrinis (2017) | " Big Food corporations have capitalized on **nutritionism**—the reduction of food’s nutritional value to its individual nutrients—as a means by which to enhance their **power** and position in global processed and packaged food markets… Through **lobbying** and participation in nutritionally focused **public–private partnerships**, they have directly sought to **inﬂuence policy and governance.** Through **market dominance** in the nutritionally enhanced foods sector, and participation in nutrition-focused rule-setting activities in agrifood **supply chains**, they have gained power to inﬂuence policy agendas. And they have used public outreach and the media to present their views on the nutritional aspects of their products, which **shapes public perceptions** and the broader regulatory environment. "  "Through market dominance in the nutritionally enhanced foods sector, and participation in nutrition-focused rule-setting activities in agrifood supply chains, they have gained power to influence policy agendas. (...) agrifood firms across the food system(...) seek to shape governance and policy" ... "Agribusiness firms can also exercise instrumental power through other means that give them access to decision-makers." |
| Collin et al. (2017) | "Food industry tactics to influence policy: philanthropy, corporate social responsibility, public relations / efforts to shape public perceptions of industry and/or its products , employing consultants, lobbying… direct **funding of politicians or parties**; creation or **funding** of alliances or front groups; funding research to create or maintain doubt about health implications; and ‘revolving door’ recruitment of key officials or politicians... litigation, trade agreements and **intimidation** of opponents" |
| de Lacy-Vawdon & Livingstone (2020) | "CDoH were described as resulting from expressions of **economic and political powe**r wielded by large corporate entities, described as “powerful economic operators”. Power imbalances were described both between corporations (large, for-profit, often trans-national entities) and governments with conflicting interests, and between corporations and individual citizens, driving behaviors that harm health."  "Groups described as being **targeted** by corporate actions within CDoH systems included: i**ndividual consumers**, groups living in vulnerable circumstances, including **children**; public h**ealth professionals** and **organizations**; **researchers** and research **organizations**; philanthropic organizations; not-for-profit organizations; special interest groups and civil society; the WHO and the UN more broadly; and government representatives. These groups were also, at times, described as promoting the interests of commerce and CDoH indirectly through their core activities."  "The dynamics constituting CDoH include broad facilitators such as **globalization** of **trade**, c**orporate structures, and regulatory system**s, articulation of social and economic **power**, **neoliberal** and **capitalist** ideologies; additional elements include corporate activities such as **marketing, corporate political activities, corporate social responsibility, extensive supply chains, harmful products and production, and issues of accessibility**. These contribute significantly to worsened global health outcomes." |
| Delobelle et al. (2016) | "In SA and elsewhere, neoliberalism, an ideology that favors deregulation, privatization, and the supremacy of markets, has strengthened the power of corporations and weakened the role of government in public health regulation."  "Business practices, activities designed to increase profits and market share, that shape the environments in which behavioral decisions are made include international trade, product **design**, marketing, retail, and **pricing.** Political practices, activities that seek to create environments in which corporations can pursue business goals without interference, include lobbying, campaign contributions, public relations, philanthropy, and sponsored research." |
| Dixon, Sindall & Banwell (2004) | "Summary of food industry tactics used to shape the food choice environment in Australia. Tactics that aim to: 1) Neutralize and pre-empt opposition through: Promoting corporate citizenship through corporate research (**sponsoring nutrition research**), Researching opposition to the industry (undermining nutritionists), Using front organizations 2) Third party endorsements (**corporate sponsorship**)(... Obtaining third party endorsement is especially important for establishing the legitimacy of industry to intervene in the regulatory environment.) through: Using professionals as media spokespeople, Using reputable bodies to advance the science supportive of industry products 3) Shaping the regulatory environment (...is critical to corporate profits) through: **Lobbying**, Providing expert advice/opinion /// **health claim** allows for a description of the relationship between a food product and its role in disease prevention. Food labelling is a significant marketing tool because of its impact on consumer confidence in food quality and the role it plays in the general discourse of diet and health. " |
| Dorfman et al. (2012) | "CSR as primarily a public relations strategy designed to achieve ‘‘innocence by association’’ as corporations align themselves with good causes to burnish their public image and protect their core business (...) CSR initiatives are often introduced when corporations fear a threat to their profitability [33]... soda industry CSR aims to position the companies, and their products, as socially acceptable rather than contributing to a social ill."  "Instead of separating moneymaking ventures from charitable donations, the contemporary soda industry CSR blurs the traditional lines between a corporation’s profit-oriented and philanthropic activities. "  "CSR focus on the consumer ('bad' behaviour and away from soda's contribution to rising obesity rates) and in their likely intent to thwart regulation... CSR campaigns focus on personal, instead of corporate, responsibility." |
| Douglas et al. (2018) | "**framing** of issues in ways that are favourable to advancing or protecting (food, soft drinks, and alcohol) industry positions and agendas… its primary goal is to maximise profit and its ultimate responsibility is to shareholders, creating inherent tensions and potential conflicts of interest"  "‘**media capture**’ is a central tactic of industry to gain influence in policy-making relating to the regulation of food and drink products." |
| Eastmure et al. (2020) | "Research on the commercial determinants of health have typically focused on the specific actions of individual businesses or industries. However, the business literature suggests that it is important to take a wider perspective and recognise that corporate entities develop broad non-market strategies to manage political, cultural and social risks to their business. Non-market strategies may include **corporate political activities (for example lobbying), Corporate Social Responsibility plans (for example, sponsorship), and legal actions against competitors and governments**. Businesses may work with others (for example through trade associations) or alone to implement their non-market strategies." |
| Fabbri et al. (2018) | Nutritional reductionism or 'Nutritionism" Commercial interests can be an important driver of this nutrient focus; food companies can indeed beneﬁt from this approach by claiming that products containing a speciﬁc **nutrient yield some health advantages**"  "food companies are often ‘rebranding themselves as nutrition companies’, offering knowledge and expertise not only in food production but also in global public health problems such as obesity. However, owing to their mission to increase proﬁts, food companies tend to frame public health problems and their solutions in ways that are less threatening to their interests (...) obesity is portrayed as a problem of poor dietary choices and lack of physical activity, and not the result of a food environment that constantly exposes people to ultra-processed drinks and foods. "  "branches located in **low- and middle-income countries**, which have been identiﬁed as **emerging markets for Big Food**. " |
| Fooks et al. (2019) | "Providing support for both h**ealth and economic-related claims**, engaging with the peer-reviewed literature, and presenting economic estimates with the appearance of a sound theoretical basis, establishes a right to be heard and taken seriously. Corporate agnogenesis then goes on to exploit the uncertainties inherent in both scientific norms and practices and economic modelling that this right of policy engagement affords. These **uncertainties** highlight the structural vulnerability of modern modes of evidence-based policy-making to corporate **agnogenesis**. The industry’s production of categorical estimates for **economic impacts**, as opposed to a range, simply reflects the political (and therefore commercial) peril in embracing uncertainty, which, inevitably, would lead to less conclusive outputs and lower estimates of effects." |
| Franz & Kickbusch (2018) | "CDoH are defined as “strategies and approaches used by the private sector to promote products and choices that are detrimental to health”. This single concept comprises a number of others: at the micro level, these include consumer and health behaviour, individualisation, and choice; at the macro level, the global risk society, the global consumer society, and the political economy of globalisation." “Large food, beverage (…) firms are among the most internationalised businesses in the entire economy. (…) Economies of scale are an important factor in the profitability of food, wholesale, retail, and beverage firms (…)” |
| Freudenberg (2012) | " Corporations use business practices such as product **design**, **advertising**, retail **distribution,** and **pricing** to achieve their goals of maximizing profit and increasing market share. Their political efforts - lobbying, campaign contributions, and public relations - seek to create a business friendly climate. In the twentieth century, consumption became a marker of modern life and corporations actively promoted the lifestyles to sustain this new order." |
| Freudenberg (2005) | "Disease promotion describes organizational practices or policies that encourage unhealthy behaviors, lifestyles, or environments. The concept is based on the social determinants of health literature, which identifies **social, political, and structural factors and processes** that contribute to patterns of health and disease." |
| Freudenberg (2018) | "...business practices such as marketing, product formulation and **pricing**, and corporate political activity such as lobbying, election campaign contributions, sponsored research, and public relations promote the behaviors, environments, and policies that shape patterns of health and disease. " |
| Freudenberg & Galea (2008)^a^ | Paraphrased: Interests from corporations and businesses that profit at the expense of public health… "consumption has replaced production as the dominant health inﬂuence of the current free market system and as the decisions of corporate managers on the **production**, **marketing**, and **distribution** of their products have become signiﬁcant determinants of health (...) Corporate practices as social determinants of health"  "Cities first emerged as agriculture produced surpluses that enabled population concentrations not directly dependent on farming." |
| Freudenberg & Galea (2008)^b^ | "... corporate practices, defined as the business and political activities of corporations. These practices result from companies' decisions about the **production**, **pricing**, **distribution**, and promotion of their products and from their political efforts to create an environment favorable for their businesses." (Health harming) choices are made in a marketplace that produces and advertises certain options and suppresses others and within a political system where certain stakeholders hold more power and influence than others." |
| Green (2019) | "Corporate interests in public health are diverse... aim to shape public health policy"  "…'harmful commodity' industries (e.g. fast food corporations) that make profits from goods which damage health… (and which have) no incentives to reduce profits through self-regulation" |
| Greenhalgh (2016) | "Market forces that shape how obesity is formed and managed" "Bias public health knowledge and practice in favour of diseases in which corporations find biovalue" "Corporation dominated structure in public health" |
| Grotz (2006) | "ILSI-sponsored research, funded by grants from companies related to the food business, as well as independent foundations, has helped to improve our collective understanding of the problem and what our next steps should be." "Supporting intervention programmes: Take 10! - focus on physical activity Kidnetic (PA and healthy eating children)- also funded by International Food Information Council (IFIC) Foundation. - 13 major food organizations as the sole sources of unrestricted grants that provided funding. America on the Move - attempts to prevent weight gain by encouraging modest changes in lifestyle, such as decreasing energy intake by 100 calories a day and increasing activity by 2000 extra steps a day."  "In addition to work within organizations such as ILSI and IFIC, some food industries have initiated their own conferences and symposia to discuss the issues and potential solutions possible within/by the food industry."  "Many food companies have also made unrestricted grants to relevant health care professional organizations in an effort to help them determine the best ways to combat obesity, including continued research" |
| Handsley & Reeve (2018) | "Transnational food companies powerfully shape the supply, demand, and consumption of food and beverage products. These companies are one of the main drivers of the increasing consumption of ultra-processed foods and sugary beverages, which are cheap, highly palatable, and sold in large portion sizes, but which are also high in energy and fat, salt and/or sugar. Transnational food companies are moving quickly into **markets in developing countries**, using strategies such as **foreign direct investment** to **increase production and sales** "  "Excess weight gain results from a complex range of individual-, family-, and community-level factors, as well as being determined by broader social, economic and cultural forces. However, the growing power of multinational food companies in the global food system is identified as a key 'vector' in the 'industrial epidemic' of obesity and non-communicable disease, ie, an epidemic emerging from the commercialisation (and widespread consumption) of health-damaging products." **These companies manufacture foods and beverages that are high in salt, fat and/or sugar, and are often promoted to children via sophisticated marketing campaigns.** Evidence suggests that unhealthy food marketing influences children's food preferences, consumption choices and dietary habits, with negative impacts on their health. Accordingly, unhealthy food marketing to children is identified as a key modifiable risk factor for childhood weight gain"  "the industry also lobbies against global and national initiatives that it sees as compromising its economic interests, raising a possibility of detrimental effects on laws and policies aimed at improving diet-related health." |
| Henriques et al. (2018) | "[The] food environment and exposure to childhood **advertising** are important causes of childhood obesity, and the concept of “obesogenic society” was coined to indicate how “environmental” factors related to food **production**, marketing and consumption are central to the problem."  "Regulatory initiatives create guidelines and/ or limits for the business sectors and strive to protect the population against abusive practices, especially the ones that originate in the corporate sector [such as] promoting voluntary agreements and self-regulation to avoid legislation that might affect their interests; influence on policy-making processes by private industry, discourse of regulation inflicting on individual's freedom; suggesting reformulation as the solution since it might impact on reducing consumption of fats, sugar and salt in the short-term, but in the long term, they might stimulate their consumption by opening new consumer markets. " (paraphrased)" |
| Herrick (2009) | "The potential causal role of the global food and drink industry (FDI), its products and practices in mounting obesity rates, has entwined discourses of blame and responsibility in complex ways, especially through the media, NGO and national governmental framing of the issue. However, to assume the responsibility needed to cultivate essential **brand value** without, importantly, attracting blame, the global FDI has turned to three strategies: ﬁrst, heavy investment in and advertisement of its health and wellness research and development efforts; secondly, continued support for its physical activity programmes, either emanating directly from companies themselves or by sponsoring state-led strategies; and thirdly, its entrance into the ﬁeld of health promotion and education in both the virtual realm and the classroom... Given the superior ﬁnancial capacity of many key FDI players relative to the state to invest in research and development, the ability of public health bodies to compete in the realms of knowledge creation, dissemination and implementation is becoming severely compromised. " |
| Ireland (2019)^a^ | "Health is not only determined by biological and genetic factors, but by the socioeconomic context of people’s lives, including income levels and educational standards. Corporate activity, such as marketing of harmful goods including unhealthy foods, tobacco, sugar-sweetened beverages and alcohol, also affects health. Commercial determinants of health are defined as “factors that influence health which stem from the profit motive. (...) Corporate activities shape our environments and determine the availability, promotion and **pricing** of consumables." |
| Ireland (2019)^b^ | "...When food corporations use commercial levers (sport is a useful vehicle for the marketing of food companies) to promote products which may be harmful to human health. " |
| Jahiel (2008) | "I propose to deﬁne a new category of **“corporation-induced diseases”** as diseases of consumers, workers, or community residents who have been exposed in the market place, work site, or community, respectively, to disease agents that are part of the products or processes of corporate activity."   " nature of for-proﬁt corporation as entities designed to maximize proﬁt for the beneﬁt of their stockholders... and to leave social and health costs for others to address." |
| Jaichuen et al. (2018) | " The food industry employs a variety and combination of CPA strategies to increase their power and influence over government policies... The two most commonly used CPA strategies were **constituency building and information and messaging**."  "The food industry plays an important role in shaping food environments and food choices. Food companies have high potential to produce a healthy food supply, but the processed food sub-sector has been criticised for driving people to desire high-calorie foods and become conditioned overeaters and for creating food environments that promote overconsumption. Evidence shows that, globally, the processed food industry is active to ensure that policy and regulatory environments are structured in their favour, and that they use their power and influence over political processes to minimize the criticisms and health concerns associated with their products. The strategies and tactics used are referred to as Corporate Political Activity (CPA)... Food industry CPA includes, among other tactics, framing the debate and shaping the evidence on diet to mould public preference in ways that favour their products, establishing relationships with policy makers, and seeking community support in various ways." |
| Jane & Gibson (2018) | "well-designed CSR strategies have been shown to be economically beneficial... profit not philanthropy is the central motivation. Public Health CSR strategies in particular have been shown to generate profit through increasing brand awareness in a target population, the creation of a health halo around a brand and **constituency building** by recruiting allies and co-opting critics... CSR can contribute to shaping public discourse and public policy in relation to individual choice and responsibility."  "ParkLives is indicative of a CSR project that aims to create a health halo around a brand and inﬂuence wider socio-ecological factors by guiding public discourse and directing opinion on the determinants of public health issues away from corporate inﬂuence and toward individual responsibility. |
| Kickbusch (2016) | “strategies and approaches used by the private sector to promote products and choices that are detrimental to health” "Breadth and depth of corporate influence is expanded as more people are reached with ever more consumption choices." |
| Knai et al. (2018) | "The strategies and approaches that unhealthy commodity industries (UCIs) (energy-dense and low-nutrient foods and beverages) use to promote their products and choices that are detrimental to health using a marketing mix: including price, **placement**, promotions, and product... These strategies include influencing the creation of evidence; questioning the effectiveness of statutory regulation and emphasizing self-regulation and public-private **partnerships**; publicly discrediting researchers; focusing on individual responsibility; attempting to frame the extent and nature of alcohol-related harms and the relevant solutions; and forming alliances with other sectors or the public to give the impression of larger support for the industry’s position" |
| Kraak, Kumanyika & Story (2009) | Paraphrased // Activities from the food, beverage and restaurant industry (commercial branding and marketing, product, place, price, promotion and product placement across diverse media) that will enhance product sales, profits and market share. If public health and corporate goals conﬂict, corporate proﬁt imperatives often will trump public health imperatives" "**Branding** is a primary objective of the integrated marketing strategy of companies, representing a name or symbol that legally identiﬁes companies, products or services that consumers differentiate among marketplace competitors." |
| Kraak et al. (2012) | "... private-sector solutions are driven by commercial imperatives to generate proﬁts, can produce inequities and may not reach vulnerable groups. Indeed, corporations have no intrinsic motivation to address existing health or social inequities that are priorities for governments and civil society. These are important tensions to acknowledge when considering a PPP approach." "NGO may be attracted by food and beverage company sponsorship and cause marketing to secure unrestricted **funds to support professional societies** and conferences, nutrition research, programmes, and sporting events. Sponsorship is a commercial activity to promote a company’s brand, not a philanthropic gift." |
| Lacy-Nichols et al. (2020) | "While the soft drink industry lobbied to promote its self-regulation, we also observed a reciprocal relationship where the ABC’s [Australian Beverages Council] self-regulation helped it to gain access to policy stakeholders and key opinion leaders in government and public health, similar to the tobacco industry’s efforts to re-establish access to policy makers by asking for ‘feedback’ on its corporate social responsibility initiatives. In addition to promoting its self-regulation to policy stakeholders, the ABC funded research that it then used to support its claim that self-regulation played an instrumental role in reducing soft drink consumption in Australia and that, consequently, further regulation was unnecessary.(...) the **timing** and **context** of the launch of self-regulation play an important role in the CPA strategy of policy substitution."  "Whereas its 1998 statement on nutrition and health emphasized why soft drinks were not part of the problem, its 2006 Commitment focused on how soft drinks were part of the solution to obesity." National codes of conduct mark the emergence of a more coordinated response to obesity and the growing unity of the food and beverage industry’s response" |
| Lauber et al. (2020) | "We confirm existing research from the national context which demonstrates that industry **framing** portrays public health issues as **individual** or lifestyle choices, and consequently interventions addressing individual responsibility, such as education, as the solution(...); our research confirms existing evidence that food industry actors support non-statutory measures and largely **oppose further statutory regulation**; respondents strongly backed a multistakeholder approach and, while acknowledging that **COI** needs to be managed, opposed the safeguards proposed by the WHO’s Nutrition department; they invoked a range of **malleable concepts**, echoing language commonly used by commercial actors globally to oppose regulation and emphasise an ostensible need for the public sector to **engage with the private sector** in policy development."  "Food industry organisations challenged the [WHO] agency’s legitimacy and mandate, firstly by framing proposed restrictions on industry engagement as incoherent with good governance principles, the SDGs, and the internationally dominant multistakeholder approach, and secondly by questioning the WHO’s mandate on NCD policy in the context of regulation... arguments around national sovereignty were employed to shift decision-making away from the WHO where industry actors did not agree with the policies proposed."  "good. The **SDGs**, by embracing multistakeholder collaboration as the way forward, set a status quo which may undermine attempts to regulate the commercial determinants of health" |
| Lencucha et al. (2018) | "NCD prevention largely hinges on healthy consumer environments, with the key risk factors for the recent rise in the incidence of NCDs being commercial products such as tobacco, alcohol and unhealthy foods." " Given that commercial interests do not always align with the public interest and in light of evidence that many of the “vectors” of NCDs are products actively promoted by industry, the regulatory role of governments must be protected from the detrimental influence of such interests. "  "Government plays a critical role in shaping food environments, through interventions that promote the economic performance and competitiveness of the agri-food sector, public health interventions that control the nature and types of foods available to consumers and interventions that seek to influence consumer food choice." "government impacts the nutritional quality of food supply and demand through the development and dissemination of information, norms, or coding systems that target both education to empower consumers to make healthy choices, and actors from the agriculture, food and health sectors that altogether define the nutritional quality of supply and demand in the food environment." |
| Lencucha & Thow (2019) | "(...) policy paradigms are a critical but often invisible underpinning of policy (in)coherence, including the a**bility of private interests to influence public policy.** In other words, the accessibility, affordability, and nature of the products in the consumer environment and the ways that companies operate within the market and in relation to government is conditioned by certain conceptions of the proper relationship between government, market and society. Specifically, we illustrate that part of the friction that inhibits healthy product policy regimes is the **persistence of the neoliberal paradigm** in shaping the relationship between market, state and society."  "One aspect of this neoliberal paradigm that we see translated into economic policy is a reluctance to impose socially oriented protections in favor of a generic rationality of economic growth. In other words, **economic growth is given greater policy importance than social goals** with the idea that growth leads to enhanced social welfare. "  "supply chains for minimally processed healthy foods such as fruit and vegetables are often characterised by significant losses, due to a s**ystemic lack of public investment** in agriculture. In contrast supply chains for highly processed vegetables, such as potato chips are highly developed due to downstream industry investment" |
| Leon & Ken (2019) | "The role that food corporations have in determining our health and nutrition is concomitant with the **power** and influence that corporations exercise across all commercial sectors. These large, powerful, and often multinational entities – collectively referred to as **Big Food** – employ a robust array of strategies to advance the organizational interests associated with a seemingly paradoxical business model: securing the continuous and ever-growing consumption of food products increasingly associated with negative health outcomes. "  "There is big money in Big Food; the **mass production** of sweet, salty, and fatty foods has proven highly lucrative, consistent with research demonstrating how corporations benefit from the exploitation of people’s biological, psychological, and **socio-economic vulnerabilitie**s in service to profit. "  "Corporations are guided by a profit-maximizing incentive structure that qualitatively differs from the organizational mandates of regulatory institutions focused on the public good... fiduciary responsibility to their shareholders is the primary organizational mandate... Additionally, successful and high-profile corporations – both past and present – generally influence the laws and regulations that govern them"  "revolving door" (...) officials of regulatory agencies who later go to work for industry have valuable knowledge and relationships that help food companies avoid regulation or have it designed in their favor. Conversely, food industry lobbyists comprise a large portion of the politically appointed positions in agencies, such as the US Department of Agriculture, that are meant to regulate and set the contract terms for food companies." |
| Lobstein et al. (2015) | "[forces shaping] the food and consumer environment, including the availability, price, and formulation of diﬀerent types of food products and the marketing practices that inﬂuence food choices and preference. "  "The food industry has a special interest in targeting children. Not only can the companies inﬂuence children’s immediate dietary preferences, but they also beneﬁt from building taste preferences and brand loyalty early in life, which last into adulthood. "  "(...)competitive nature of food multinationals and their **rapid penetration of low-income and middle-income markets** (...) seeking to expand their markets for (...) highly processed foods and sweetened beverages, which contain ingredients that condition the taste buds of children to a lifetime of consuming energy-rich and nutrient-poor products. "  "The rise in the promotion of soft drinks shows the forces underlying the nutrition transition; the move from traditional food supplies to food supplies strongly inﬂuenced by mass produced, branded, and highly promoted products. "  "Economic investments embedded in the creation and perpetuation of sales of food to children suggests that the debate over policies to prevent child obesity is primarily a political argument about competing economic and health interests. Proposals for controlling food environments, and especially food marketplaces, are likely to be strongly resisted, as they have been with alcohol and tobacco policy making. "  "Agriculture, food supply, and food environment targets, are needed in order to improve nutritional health; increased involvement of governments will be needed to achieve such targets … a closer look at the forces shaping food supplies is needed to tackle both undernutrition and overnutrition"  "Agricultural production and food supply targets need to be set by international governmental agencies without conflicted economic interests, with independent monitoring, and with the actions of all participants held to account." |
| Maani-Hessari et al. (2019) | "“corporate” or “commercial” determinants of health: strategies, approaches, and influences from (health-harming product) manufacturers" "Corporate determinants of health - how manufacturers of harmful products and activities use similar language and tactics when faced with policies that threaten their profits." |
| Madureira-Lima & Galea (2019) | "Commercial interests have long been identiﬁed as a macrosocial determinant of health."  "Corporate permeation refers to the extent to which corporations penetrate all aspects of society, from macrosocial and political aspects, such as corporate donations to **election campaigns,** to shaping individual consumption patterns, through, for example, **advertising** that encourages eating calorie-dense, nutrition poor foods at fast food chains. "  “Vehicles of [Corporate or Commercial] Power”- the Political Environment, Preference Shaping, Knowledge Environment, Legal Environment, and Extra Legal Environment- and the corresponding “Practices of Power”—the tactics that enable corporate power"  "The US Department of Agriculture not only receives financial contributions from agribusiness, but also, historically, has had staff and agriculture committee members from the farming community" |
| McKee & Stuckler (2018) | "In **2013**, Millar coined the term “corporate determinants of health.” He described how some companies acted in ways that promoted health... (while others) employing the language of corporate social responsibility, pursued proﬁt above all else, marketing unhealthy products, exploiting workers and suppliers, and giving nothing back to society. "  "In **2016** Kickbusch identiﬁed four channels through which inﬂuence was exerted: **marketing, lobbying, corporate social responsibility** strategies to “whitewash tarnished reputations,” and extended supply chains. "  "We trace the development of the concept of the corporate determinants of health. We argue that these determinants are predicated on the unchecked power of corporations and that the means by which corporations exert power is increasingly unseen (...) An effective response to the corporate and commercial determinants of health must address the **power imbalance** between global corporations, which are accountable only to their owners and shareholders, and governments, which are accountable to their citizens. "  "We identify four of the ways corporations inﬂuence health: deﬁning the dominant narrative; setting the rules by which society, especially trade, operates; commodifying knowledge; and undermining political, social, and economic rights. |
| McKee, Steele & Stuckler (2019) | "the commercial determinants of health” pays particular attention to the hidden and invisible forms of power, whereby large corporations use various methods to shape thinking about what are appropriate responses to the health consequences of their products." |
| Mialon (2020) | "Corporations seek to make a profit from their commodities. They use ‘**business** practices’ to run their activities; and ‘**market** practices’ to develop, produce and sell their commodities. Corporations also use **political** practices to secure a favourable policy environment."  "Specifically, the business practices of corporations include, amongst other things, the control of the supply chain and market concentration (through mergers and acquisitions for example); labour practices; taxation payments and profits shifting; and the privatisation of utilities. (...) Corporations also use market practices such as product research and development; pricing; marketing, including advertising and retail distribution. (...) The political influence of corporations is also known as ‘corporate political activity’ (CPA)" |
| Mialon et al. (2019) | Paraphrased: "Proﬁtability is the ultimate driver of industry practices." "Activities and practices from the food industry (farmers, manufacturers, fast food restaurants, the collective catering sector, retailers, and their trade associations) that allow them to protect their profits from the manufacture, promotion and, sale of ultra-processed food and drink products (UPF) (...) The food industry can use their political and economic power (corporate political activity) to delay, weaken or prevent the development of effective public health policies to reduce the consumption of UPF, if they jeopardise their market share and profitability. " |
| Mialon & Mialon (2018) | "Critical social scientists try to reduce illusions and avoidable suffering in the population(18). For the present study, the illusion was that the food industry is merely an economic actor, while there is evidence that it also engages in political activities. We considered that the food industry, which sells and markets some products that may be considered as unhealthy, has an inherent conﬂict of interest in the development of public health policies and programmes that would restrict its activities. The political practices of the food industry were therefore considered to be a potential determinant of health. "  "Through the **‘information and messaging**’ strategy, the food industry selectively produces and disseminates information that would be beneﬁcial to its activities, to inﬂuence public policies and public opinion in ways favourable to its companies - ﬁve different types of practices in that strategy: (i) **lobbying**; (ii) stressing the economic importance of the industry; (iii) promoting deregulation; (iv) **framing the debate** on diet- and public health-related issues; and (v) **shaping the evidence** base on diet- and public health-related issues." **"Constituency building**... attempts to inﬂuence public opinion and public policies and programmes. " "**Policy substitution** When at risk of having its products regulated, the food industry has tried to propose alternatives, such as self regulation and voluntary codes of conduct."  "**‘Opposition fragmentation and destabilisation**’ is a strategy described in the literature when a company attacks individuals or groups of individuals known to be sceptical of the company’s products, practices or policies(9). " |
| Mialon, Swinburn & Sacks (2015) | " The food industry has been identiﬁed as a vector of disease, through their supply of unhealthy food products, their marketing strategies and their corporate political activity (CPA; a term used here to refer to ‘corporate attempts to shape government policy in ways favourable to the ﬁrm’ ... The dominance, in terms of market share, of prominent food companies in many countries, coupled with their collective efforts as part of trade associations, has meant that a small number of companies have a large degree of economic power, which readily translates to political power. Accordingly, there is a heightened risk that food industry proﬁts will be privileged above other considerations, resulting in food governance and public health policy that does not adequately balance public and commercial interests." |
| Millar (2013) | "(...) selling products that are damaging to health and the environment, at prices that do not account for these damaging effects (**externalities**) and often target consumers that are ill-informed and susceptible (e.g., children). These include businesses involving tobacco, alcohol, drugs, junk foods and beverages, resource extraction, arms production and the electronic media. "  "(...) bad corporations which simply take on CSR as a means of raising their profile and offsetting some of the damage they are doing. (...), selling products at prices that are far below market value because they have been allowed to shift responsibility for the negative effects of their products (so-called “externalities”) to society (...), contributing to growing economic and health inequities. "  "…activities include the **sponsorship** of sports and cultural events and **school** activities that are really **advertising** opportunities in disguise. And these corporations often go to great lengths to avoid paying taxes and expend much time and money to **lobby** for **subsidies** and deregulation." |
| Miranda et al. (2019) | "Economic improvement**, trade liberalization** and increasing **urbanization** have resulted in important changes in the LMIC food environment, defined as the **composition, promotion, availability, accessibility and affordability of foods**. In recent years, LMIC settings have experienced an increased **availability of foods produced** by large international food corporations, as well as an important **expansion of supermarkets and fastfood chains** driven in part by the so-called commercial determinants of health. As a result, people living in urban areas have **rapid and cheap access to more packaged, ready‐to‐eat or ready‐to-heat foods** manufactured by multinational food corporations, as compared to local foods that could be obtained in traditional open air markets, such as fruits, vegetables and cereals. In the case of children, this scenario is worsened by the presence of street vendors in the surroundings of daycares and schools who now offer snacks and industrialized beverages to children instead of natural foods, as shown in examples from Brazil, Guatemala and Mexico. Compounding this, an important proportion of children do not have access to clean water during long periods in the day, and may be offered sugar-sweetened beverages by preference. Similarly, over the past two decades, away-from-home food intake has undergone a large increase, with, for example, fast-food chains now spread all over Latin America."  "Moreover, recent evidence indicates that the nutrient c**omposition of packaged food products varies** substantially around the world, being considerably less healthy in LMICs such as Chile and Mexico."  "The growth in the intake of snacks, soft drinks and processed foods in LMICs is also faster than that in HICs, and is projected at 20% in the next 5 years, whereas little or no growth is expected in HICs."  "The rise in the consumption of unhealthy commodities reflects the fact that multinational companies are increasingly targeting LMICs not only for their huge collective population size, but also because governmental legislation protecting LMIC populations from unhealthy commodities remains much weaker than in HICs, where the impacts on health are already well recognized and supported by a strong scientific evidence base." |
| Molnar (2005) | "The soaring increase in obesity and type 2 diabetes among children is a public-health crisis, plausibly linked to the 'toxic environment' created in large part by the food industry" [6]. (One) trend is the intensified focus on **schools as marketing** venues for corporations (school **commercialism**), particularly those that sell nutritionally dubious products, including salty snacks, fat-laden foods, and sugary soft drinks. " |
| Moodie et al. (2013) | " The term industrial epidemic13,14 has been used to describe health harms associated with various goods including... the food and drink industries.14 In industrial epidemics, the vectors of spread are not biological agents, but transnational corporations. Unlike infectious disease epidemics, however, these corporate disease vectors implement sophisticated campaigns to undermine public health interventions. (biasing research findings, co-opting policy makers and health professionals, **lobbying** politicians and public officials to oppose public regulation and by encouraging voters to oppose public regulation)." |
| Moodie et al. (2006) | " (a) fundamental driver underpinning the rise in obesity is the power of commerce, and that this represents a clear ‘market failure’ to deliver optimal personal preferences. (...) If the market determines our choices and these choices result in outcomes that are detrimental to health (e.g. obesity) and fail to sustain and promote social and individual goals, then the market has failed."  "Obesity has multiple levels of determinants (...) some of (which) are related to commercial products. (...) The combination of high levels of promotion, widespread availability and low prices of these products (e.g. HFSS, energy-dense, low-nutritious foods) (...) overwhelmingly drive the behaviours in the direction of positive energy balance (promoting obesity)"  "Some agricultural policies in the EU create an obesogenic food supply whereby oil-producing plants are subsidised but fruit and vegetable productions are regulated to maintain high prices" |
| Nestle (2000) | " **Pouring rights** contracts ...compromise nutritional principles for financial reasons … The well-financed promotion in schools of soft drinks and other foods of poor nutritional quality directly undermines federal efforts to improve the dietary intake of children and reduce rates of obesity" "... exclusive rights to sell one brand are the latest development in the increasing commercialization of school food. These contracts, intended to elicit brand loyalty among young children who have a lifetime of purchases ahead of them" “Examples of methods used by soft drink companies to market their products to children in and outside school [include] coupons for fast food, (…) discount cards, coupons.”   "commercial interests that contribute to poor nutrition." "From its inception, the purpose of the School Lunch Program was to improve the nutritional status of children while providing an outlet for surplus agricultural commodities. "  "Creating programmes (i.e. School Lunch Programmes) to improve nutritional status of vulnerable populations (i.e. children) while providing an outlet for surplus agricultural commodities" |
| Olstad & Raine (2013) | "the food industry controls the food supply, and not only responds to but actively shapes consumer demand for its products through marketing… given the preponderance and extensive marketing of unhealthy foods in recreational facilities, it is difficult to argue that the current environment supports free and independent food purchasing decisions." |
| Panjwani & Caraher (2014) | "The Responsibility Deal legitimises industry involvement in the design of policy measures, with no sanctions or targets to ensure those measures drive public health goals. It has afforded private interests the opportunity to influence in their favour the public health policies and strategies that affect their products." |
| Powell (2016) | " Healthy lifestyles education programmes represent a new ‘brand’ of health, health education and corporation. The child citizen is governed to become the child-consumer. Corporations’ anxieties about being blamed for childhood obesity are fused with technologies of ‘healthy consumption’: the consumption of corporate products, corporate philanthropy, the corporate brand and corporate ‘education’(...) **Product placement** is a tactic to attach a corporate product to children’s knowledge of what a healthy product looks like and which speciﬁc products they needed to consume." |
| Raine (2013) | " ‘commercial activities in schools’ is defined here as those business practices in schools that implicitly or explicitly advertise or market products/services to pupils, test products on children, or promote a company, its agenda or viewpoint on particular issues. " "Direct advertisement (...) also involves the distribution to pupils of free samples, coupons and gifts containing advertising" |
| Schram, Labonté & Sanders (2013) | "The very existence of a global food industry has vastly changed what types of foods have become available. The overriding responsibility of transnational food corporations (TFCs) to their stakeholders requiring that they maximize investor returns, usually by increasing profits, gives industry only two options; push more product or improve profit margins. The most profitable items in this industry are highly processed products, including fast food, snacks, and beverages that are made up of large amounts of starch, sugars and low-quality fats (...) the two revenue streams of TFCs, more product and more profitable products, have contributed to rising rates of overweight and obesity worldwide."  "...dietary shift is also attributed to the continued agricultural export **subsidies** that allow developed countries to artificially supress food prices making it difficult for domestic markets in **developing countries** to complete" |
| Schrempf (2014) | "FF chains are socially connected to obesity through their business activities and products… FF chains are increasingly regarded as one actor having a responsibility for obesity, thereby being regarded as political actors." |
| Smith, Buse & Gordon (2016) | "7) Demanding action to address commercial determinants of (ill) health (to achieve SDGs) - how to protect health when trade agreements grant harmful industries access to markets around the world, decisions made in a corporate office in one country affect health outcomes in another, and the commercial marketing of harmful substances pervasively crosses borders. [...] Aiming to address threats posed by the commercial junk food industry “the governance of food **production** and **distribution** cannot be left to economic interests alone”. [...there is a need to] advocate action on the commercial determinants of poor diets such as: regulations to reduce children’s exposure to marketing, compositional limits on saturated fat, sugar and sodium content; and that all trade an investment policies be assessed for their potential health impacts" |
| Sonntag et al. (2015) | "There are several factors that have been identiﬁed in the literature as potential determinants of the growth of childhood overweight and obesity… individuals interact with multiple micro environments or local settings such as schools, homes and food retailers, that involve food, physical activity or both (which) can contribute to overweight and obesity by encouraging unhealthy diets in terms of increased consumption of energy-dense, nutrient-poor food and beverages.  “Microenvironments are in turn inﬂuenced by broader macroenvironments and actors such as health systems, the food industry and government policies, which are often beyond the control of individuals... As one of the many components of the macro environment, food promotions inﬂuence children’s preferences, consumption and purchasing requests to parents. "  "food industry uses persuasive marketing techniques such as attractive product packing, toys, and emotional **appeals** to forge long-lasting relations with children and create brand loyalty in the short and long run " |
| Stuckler & Nestle (2012) | "Growth in profit is the primary goal of corporations... Big Food attains profit by expanding markets to reach more people, increasing people’s sense of hunger so that they buy more food, and increasing profit margins through encouraging consumption of products with higher price/cost surpluses. Industry achieves these goals through food **processing** and marketing." |
| Stuckler et al. (2012) | "Neoliberal policies, including the opening of markets to trade and foreign investment, create environments that are conducive to the widespread **distribution** of unhealthy commodities by multinational firms… consumption choices and habits are increasingly affected by shifts in food type, price, availability, and marketing that favour unhealthy commodities." "**Foreign direct investment** increases risks of rising unhealthy commodities among **LMIC**s" |
| Swinburn (2008) | "the biggest obesogenic environmental change has been the increased availability and promotion of cheap, energy-dense foods."  "The driving forces behind the consumption, indeed overconsumption, of these obesogenic products are commercial (profit incentives), and market economies are now the backbone of all successful economic systems. A high consumption constitutes a 'commercial success' (...) (however) The increasing obesity prevalence and inequalities can, therefore, be described as a 'market failure' because the free market system is failing to promote and sustain long-term individual and social goals." |
| Swinburn et al. (2019) | "Industries with vested interests, such as transnational food and beverage manufacturers, are powerful and highly resourced **lobbying** forces that have opposed governments' attempts to regulate commercial activities or modify them through fiscal policies, such as imposing a tax on sugary drinks or changing **agricultural subsidies**. Politicians are either **intimidated** by industry opposition or they might hold beliefs that education and maket-based solutions that are grounded on **neoliberal** economic and governance models are sufficient to reverse the obesity epidemic... [and there is no] sufficient public demand for action to overcome the industry opposition and government reluctance (policy inertia)."  "In economic systems in which the vested interests of powerful transnational corporations produce financial benefits that are maximally privatised, the social and environmental costs or **externalities** fall to consumers, taxpayers, ratepayers, and future generations. The major risks to society and economic development in the future are heavily neglected." |
| Thorn (2018) | "While social determinism is an important framing for public policy development and discussion it lacks something when it comes to power and politics. Perhaps a better framing in the debate about remedying **social and economic inequality** is to think about commercial determinants of health, and their partners in crime—addictive or unhealthy commodity industries. “Commercial” because it speaks to power. It speaks to the power of the corporation, political influence, money, wheeling and dealing, and often corruption. “Social” does not, it generally speaks to weakness and disadvantage. "  "Neo- liberal, market- oriented economies have tended to acquiesce to corporate power. Competition policy, deregulation, privatisation and outsourcing have strengthened the hand of corporations and increased the risks of perceived and real undue influence on the political class." |
| Weishaar et al. (2016) | "In order to maintain positive public and political perceptions of their activities, advance their business and political goals, and influence public and political debates, different industries (e.g. food and soft drink corporations) employ similar political strategies and practices... A key corporate strategy is framing public and political debates to align with commercial interests. Framing involves the generation of beliefs and ideas that provide a structure for thinking about issue" |
| Wells (2013) | "The public’s **behavioural motivations** in relation to dietary intake and physical activity patterns are based on factors such as **necessity, availability, price, convenience and enjoyment**. However, these motivations are not ‘neutral’, but have rather been heavily inﬂuenced by a wide range of commercial companies... The construction of the modern consumer is a complex process, involving the promotion both of new aspirations, and of manipulative practices… The global obesity epidemic is therefore driven by the increasing commercial constraint of people within the obesogenic niche...  ‘Obesogenic’ food companies maximize their proﬁts by maintaining or increasing sales (e.g. increased palatability and shelf-life, and by being cheap promoting large profit-margins), and prioritizing both types of addictive mechanism (e.g. 'value deals' and addictive properties of sugar, salt, fat and caffeine on foods)."  "“(…)the economic causes of under-nutrition and over-weight have a common structural basis, driven by multinational corporations’ demand for cheap labour and new consumers.” |
| West & Marteau (2013) | “Factors that inﬂuence health which stem from the proﬁt motive.”  "(Activities from) all those industries that provide goods or services that many people want, or can be induced to want (...) (and that), the production and consumption of all these goods comes at a huge cost, including global health and climate change." |

# 5. Table S5. Data-driven themes, subthemes and mechanisms

| **Spheres of action (n=3)**  **“Data-driven themes”** | **Strategies**  **(n=12)**  **“Themes”** | **Practices**  **(n=26)**  **“Subthemes”** | **Mechanisms**  **(n=85)** |
| --- | --- | --- | --- |
| 1) Political and legal | 1.1) Framing the evidence and debate | a) Shaping narrative and debate of health and disease | Framing political debate (e.g. nanny state discourse) |
|  |  | (Frame obesity as an issue of individual and societal choices and responsibilities) | Suggesting market-based / educational solutions |
|  |  |  | Nutritionism (i.e. reductive focus on individual nutrients, in isolation from the foods and diets in which we ﬁnd them) |
|  |  |  | CSR Creating health halo effect (i.e. deflect criticism from health damaging products) |
|  |  |  | Media capture |
|  |  |  | Suggesting industry is part of the solution (e.g. strategic discourse, self-regulatory measures like reformulation of products, partnerships) |
|  |  |  |  |
|  |  | b) Creating evidence | Funding research, conferences and evidence for obesity causes and solutions |
|  |  |  | Claiming to be the experts in nutrition  Agnogenesis (production of information or ideas that create ignorance or doubt beyond that merited by empirical evidence) |
|  |  |  |  |
|  | 1.2) Influencing governance of food production, trade and investment towards a more liberalised trading environment globally | a) Participating in foreign direct investment (FDI) practices | Corporate economic/political power allows unrestricted international capital flows |
|  |  |  | Accelerated penetration of domestic markets through FDIs |
|  |  |  | Growth of transnational food companies (company mergers, acquisitions and joint ventures) |
|  |  |  | Becoming a primary source of external financing in developing countries |
|  |  |  | Increased control over all levels of food system (production, processing, distribution and retail) |
|  |  |  |  |
|  |  | b) Taking advantage of neo-liberal economic policies that favour  trade liberalisation and globalisation | Bringing international food imports into domestic markets at lower prices due to reduction in import barriers |
|  |  |  | Using taxation minimisation strategies and tax havens - no international organisation overseeing the full profit report from a transnational company |
|  |  |  | Market integration into global food markets through structural adjustment programmes (SAPs) to restore economic balance and repay international loans  Privatisation of state-run companies  Usage of trade, loans and aid to ensure dependency |
|  |  |  |  |
|  |  | c) Revolving door between regulatory agencies – food and agriculture industries | Officials from regulatory agencies who move on to work for industry giving access to decision-makers to help food companies avoid regulation and set the contract terms. |
|  |  |  | Regulatory officials being recruited from food and agriculture industries to then return to businesses like lobbying firms |
|  |  |  | Regulation agencies receiving financial incentives from agri-business |
|  |  |  | Using their market dominance to set the rules of the agri-food supply  chains, they have gained power to influence policy agendas. |
|  |  |  | Agri-business creating nutrition focused partnerships (e.g. Scaling Up Nutrition movement – SUN) |
|  |  |  |  |
|  | 1.3) Influencing policymaking process | a) Lobbying | Influencing policymakers to maintain a business-friendly regulatory environment |
|  |  |  |  |
|  |  | b) Constituency building | Revolving doors' (i.e. enabling ofﬁcials to move into lucrative consultancies once they have retired) |
|  |  |  | Forming alliances with key opinion leaders, policymakers, health professionals to support industry position. |
|  |  |  | Encouraging voters to oppose public regulation |
|  |  |  | Philanthropic activities, PPPs, PR and CSR by recruiting allies and co-opting critics |
|  |  |  |  |
|  |  | c) Funding key stakeholders / opinion leaders | Election campaigns |
|  |  |  | Health and nutrition organisations |
|  |  |  | Opposition groups |
|  |  |  |  |
|  |  | d) Intimidating opposition | Threaten with legal action |
|  |  |  |  |
|  | 1.4) Limiting corporate liability | a) Externalising costs using unregulated areas of activity | Externalising occupational health, environmental and social costs  Keeping prices artificially low by outsourcing sectors of the business  Shifting profits to tax havens |
|  |  |  |  |
| 2) Production, processing and design | 2.1) Reducing processing/manufacturing costs | a) Optimising food manufacture and processing | Mass production and economies of scale (cost minimisation)  Long shelf-life ingredients (HFSS, additives)  Ultra/processing (reducing nutritional value of foods) |
|  |  |  |  |
|  |  | b) Reformulating products | Changing formulas of energy-dense, nutrient-poor foods instead of finding ways to replace them altogether (e.g. reduce fat, reduce sugar, enrich products with vitamins and minerals) |
|  |  |  |  |
|  |  | c) Increasing product appeal with low cost ingredients | Cheap ingredients that enhance palatability (e.g. sugar and fat)  Flavour enhancing ingredients - addictiveness (e.g. HFSS and caffeine) |
|  |  |  |  |
|  | 2.2) Increasing market share  (more sales, more revenue, more money to invest, expand and optimise production practices) | a) Strengthening penetration in emerging markets | Glocalisation – design products according to local ingredient offer & demand |
|  |  |  | Diversification of product portfolio |
|  |  |  | Company mergers |
|  |  | b) Taking advantage of cheaper production costs in emerging markets | Bulk buying of local commodity production at lower prices |
|  |  |  | Settling production / processing plants in places where labour is cheaper |
|  |  |  |  |
|  | 2.3) Agribusiness food/ ingredient supply | a) Benefiting from agricultural  subsidies | Conversion of traditional domestic production to export-oriented production or cash-crops |
|  |  |  | Subsidies for crops that are economically relevant (e.g. sugar in the global south) |
|  |  |  | Creation of import dependency - developed countries can keep prices artificially low, making it difficult for domestic markets |
|  |  |  |  |
|  |  | b) Prioritise commodities with profitable by-products (e.g. corn, soybean) | Increased per-acre productivity drive a reduction in prices which stimulate further productivity and profits (e.g. corn, soybeans) |
| 3) Marketing and preference shaping | 3.1) Promotion to increase brand awareness and visibility | a) Advertising | Targeted / integrated marketing |
|  |  |  | Neuromarketing (i.e. understanding consumers choices and using triggers to elicit emotions - Coca-Cola selling joy) |
|  |  |  | Focus on selling new aspirations rather than the product itself |
|  |  |  |  |
|  |  | b) CSR, sponsorship and branding | Sports, cultural and scientific events (e.g. conferences) |
|  |  |  | Educational materials and school activities |
|  |  |  | Government health programmes and professional societies |
|  |  |  | CSR increase brand awareness in targeted populations |
|  |  | c) Partnerships | Corporate philanthropy |
|  |  |  | Public-private partnerships (PPPs) |
|  |  |  |  |
|  | 3.2) Influencing consumers perceptions of product (real vs aspiration) | a) Packaging products with “added value” claims | "Healthy" line of products from same company (e.g. ‘sugar free’, ‘fat free’, ‘gives you energy) |
|  |  |  | “Delux” line of products |
|  |  |  | Appealing toys and characters |
|  |  |  | Attractive colours of packaging |
|  | 3.3) Creating brand loyalty | a) Pouring rights  (exclusive permission for a beverage manufacturer or bottler to control distribution and sales in a venue) | Recreational facilities |
|  |  |  | Stadiums |
|  |  |  | Schools |
|  |  |  |  |
|  |  |  | Corporate creation of curriculum materials |
|  |  | b) Commercialising education | Sponsoring educational materials |
|  |  |  | Incentive programmes (i.e. completing established quotas in exchange for products, money, goods or services) |
|  |  |  | Exclusivity agreements |
|  |  |  | Sponsorship of programmes and activities |
|  |  |  | Fund-raising |
|  |  |  | Appropriation of space, branded infrastructure (sports facilities, lunchrooms, scoreboards) |
|  |  |  | Privatisation |
|  |  |  |  |
|  | 3.4) Product placement and distribution | a) Ubiquitous presence of  processed foods | Increased product availability |
|  |  |  | Increased product accessibility |
|  |  |  | Convenience of products |
|  |  |  | Extended supply chains |
|  |  |  |  |
|  | 3.5) Pricing | a) Cheap selling price | Affordability - cheap energy-dense foods products |
|  |  |  | Portion size – bigger portions cheaper price |
|  |  |  |  |
|  |  | b) Price promotions | Discounts, bundle and volume deals |
|  |  |  | Online promotions |
|  |  |  | Printed coupons |
|  |  |  | Trade promotions |
|  |  |  | Reward programmes |

# Table S6. Prisma 2009 Checklist

| 1. **Section/topic** | **#** | **Checklist item** | **Reported on page #** |
| --- | --- | --- | --- |
| **TITLE** | | |  |
| Title | 1 | Identify the report as a systematic review, meta-analysis, or both. | 1 |
| **ABSTRACT** | | |  |
| Structured summary | 2 | Provide a structured summary including, as applicable: background; objectives; data sources; study eligibility criteria, participants, and interventions; study appraisal and synthesis methods; results; limitations; conclusions and implications of key findings; systematic review registration number. | 2 |
| **INTRODUCTION** | | |  |
| Rationale | 3 | Describe the rationale for the review in the context of what is already known. | 3 and 4 |
| Objectives | 4 | Provide an explicit statement of questions being addressed with reference to participants, interventions, comparisons, outcomes, and study design (PICOS). | 4 |
| **METHODS** | | |  |
| Protocol and registration | 5 | Indicate if a review protocol exists, if and where it can be accessed (e.g., Web address), and, if available, provide registration information including registration number. | 4 |
| Eligibility criteria | 6 | Specify study characteristics (e.g., PICOS, length of follow-up) and report characteristics (e.g., years considered, language, publication status) used as criteria for eligibility, giving rationale. | 4 and 5 |
| Information sources | 7 | Describe all information sources (e.g., databases with dates of coverage, contact with study authors to identify additional studies) in the search and date last searched. | 4 |
| Search | 8 | Present full electronic search strategy for at least one database, including any limits used, such that it could be repeated. | Supporting information section 1. Search strategy |
| Study selection | 9 | State the process for selecting studies (i.e., screening, eligibility, included in systematic review, and, if applicable, included in the meta-analysis). | 4 and 5 |
| Data collection process | 10 | Describe method of data extraction from reports (e.g., piloted forms, independently, in duplicate) and any processes for obtaining and confirming data from investigators. | 5 |
| Data items | 11 | List and define all variables for which data were sought (e.g., PICOS, funding sources) and any assumptions and simplifications made. | 5 and 6 |
| Risk of bias in individual studies | 12 | Describe methods used for assessing risk of bias of individual studies (including specification of whether this was done at the study or outcome level), and how this information is to be used in any data synthesis. | 6 |
| Summary measures | 13 | State the principal summary measures (e.g., risk ratio, difference in means). | NA |
| Synthesis of results | 14 | Describe the methods of handling data and combining results of studies, if done, including measures of consistency (e.g., I^2^) for each meta-analysis. | 6 |

Page 1 of 2

| **Section/topic** | **#** | **Checklist item** | **Reported on page #** |
| --- | --- | --- | --- |
| Risk of bias across studies | 15 | Specify any assessment of risk of bias that may affect the cumulative evidence (e.g., publication bias, selective reporting within studies). | 6 |
| Additional analyses | 16 | Describe methods of additional analyses (e.g., sensitivity or subgroup analyses, meta-regression), if done, indicating which were pre-specified. | NA |
| **RESULTS** | | |  |
| Study selection | 17 | Give numbers of studies screened, assessed for eligibility, and included in the review, with reasons for exclusions at each stage, ideally with a flow diagram. | 7 & Fig 1. Prisma flow diagram |
| Study characteristics | 18 | For each study, present characteristics for which data were extracted (e.g., study size, PICOS, follow-up period) and provide the citations. | 8 & Online Supplementary Table S2. Descriptive table of the 81 included articles p4 |
| Risk of bias within studies | 19 | Present data on risk of bias of each study and, if available, any outcome level assessment (see item 12). | 8 & Online Supplementary Table S2 p4 and Table S3. p10 |
| Results of individual studies | 20 | For all outcomes considered (benefits or harms), present, for each study: (a) simple summary data for each intervention group (b) effect estimates and confidence intervals, ideally with a forest plot. | 10 & Online Supplementary Table S2. p4 and Table S4. Extracted quotes for definitions and mechanisms p18 |
| Synthesis of results | 21 | Present results of each meta-analysis done, including confidence intervals and measures of consistency. | 9 to 23; Table S5. Data-driven themes, subthemes and mechanisms p37 |
| Risk of bias across studies | 22 | Present results of any assessment of risk of bias across studies (see Item 15). | 8 |
| Additional analysis | 23 | Give results of additional analyses, if done (e.g., sensitivity or subgroup analyses, meta-regression [see Item 16]). | NA |
| **DISCUSSION** | | |  |
| Summary of evidence | 24 | Summarize the main findings including the strength of evidence for each main outcome; consider their relevance to key groups (e.g., healthcare providers, users, and policy makers). | 24 to 27 |
| Limitations | 25 | Discuss limitations at study and outcome level (e.g., risk of bias), and at review-level (e.g., incomplete retrieval of identified research, reporting bias). | 25 and 26 |
| Conclusions | 26 | Provide a general interpretation of the results in the context of other evidence, and implications for future research. | 27 |
| **FUNDING** | | |  |
| Funding | 27 | Describe sources of funding for the systematic review and other support (e.g., supply of data); role of funders for the systematic review. | 28 and 29 |

*From:*  Moher D, Liberati A, Tetzlaff J, Altman DG, The PRISMA Group (2009). Preferred Reporting Items for Systematic Reviews and Meta-Analyses: The PRISMA Statement. PLoS Med 6(7): e1000097. doi:10.1371/journal.pmed1000097

For more information, visit: **www.prisma-statement.org**.
